# Supplementary material for: A randomized, double-blind, placebo-controlled pilot trial of low-intensity pulsed ultrasound therapy for refractory angina pectoris
Source: PLoS One. 2023 Jun 23;18(6):e0287714. doi: 10.1371/journal.pone.0287714 (PMC10289346; doi:10.1371/journal.pone.0287714)

Appendix Figure A

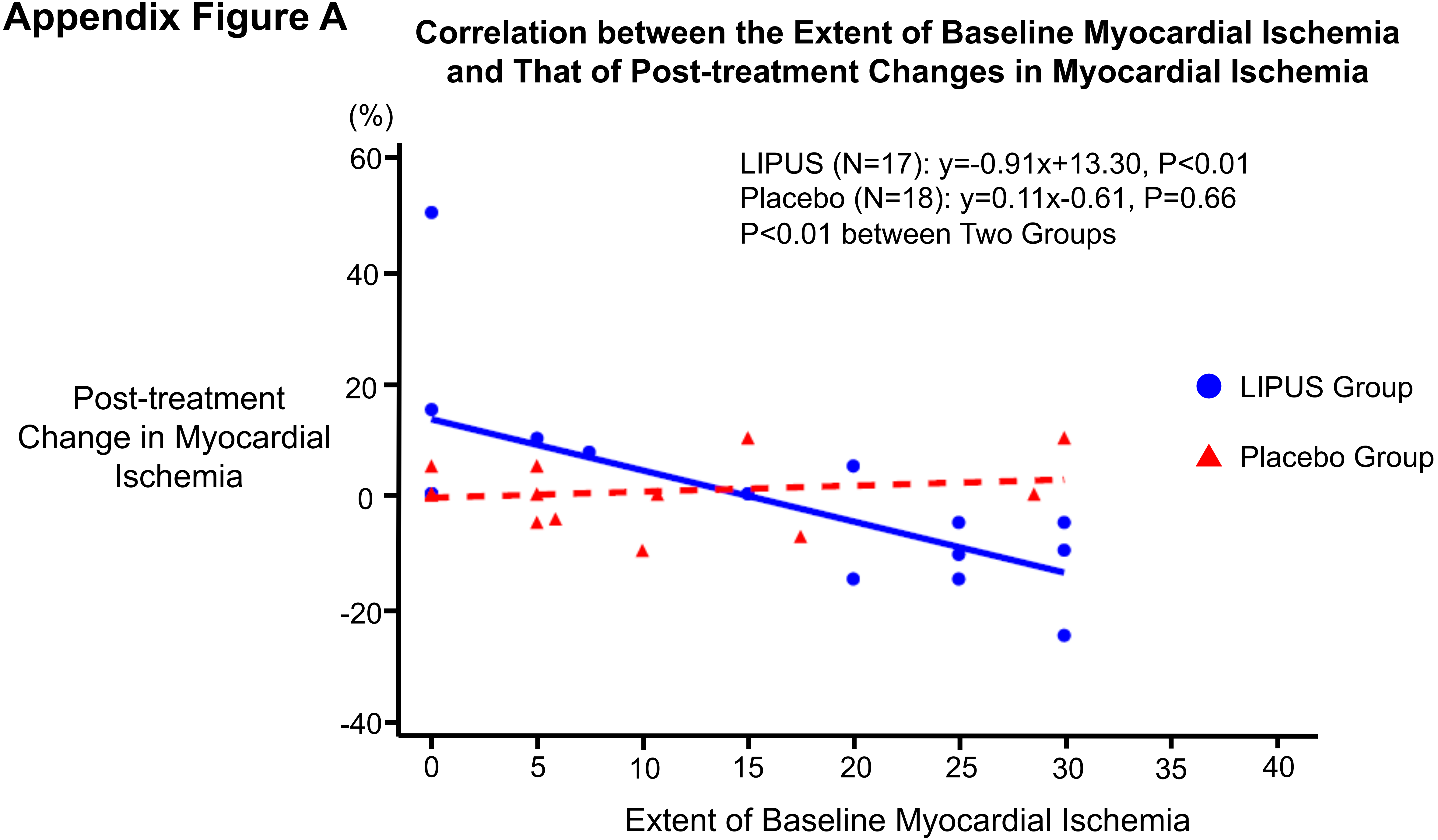

Supplement: S1 Fig — Myocardial perfusion images in a representative case in response to the LIPUS therapy. (PDF) [file pone.0287714.s003.pdf]
