# Supplementary material for: A randomized, double-blind, placebo-controlled pilot trial of low-intensity pulsed ultrasound therapy for refractory angina pectoris
Source: PLoS One. 2023 Jun 23;18(6):e0287714. doi: 10.1371/journal.pone.0287714 (PMC10289346; doi:10.1371/journal.pone.0287714)

Appendix Figure B

Representative Images of a Responder Patient in Stress Myocardial Perfusion Imaging

Before LIPUS Therapy

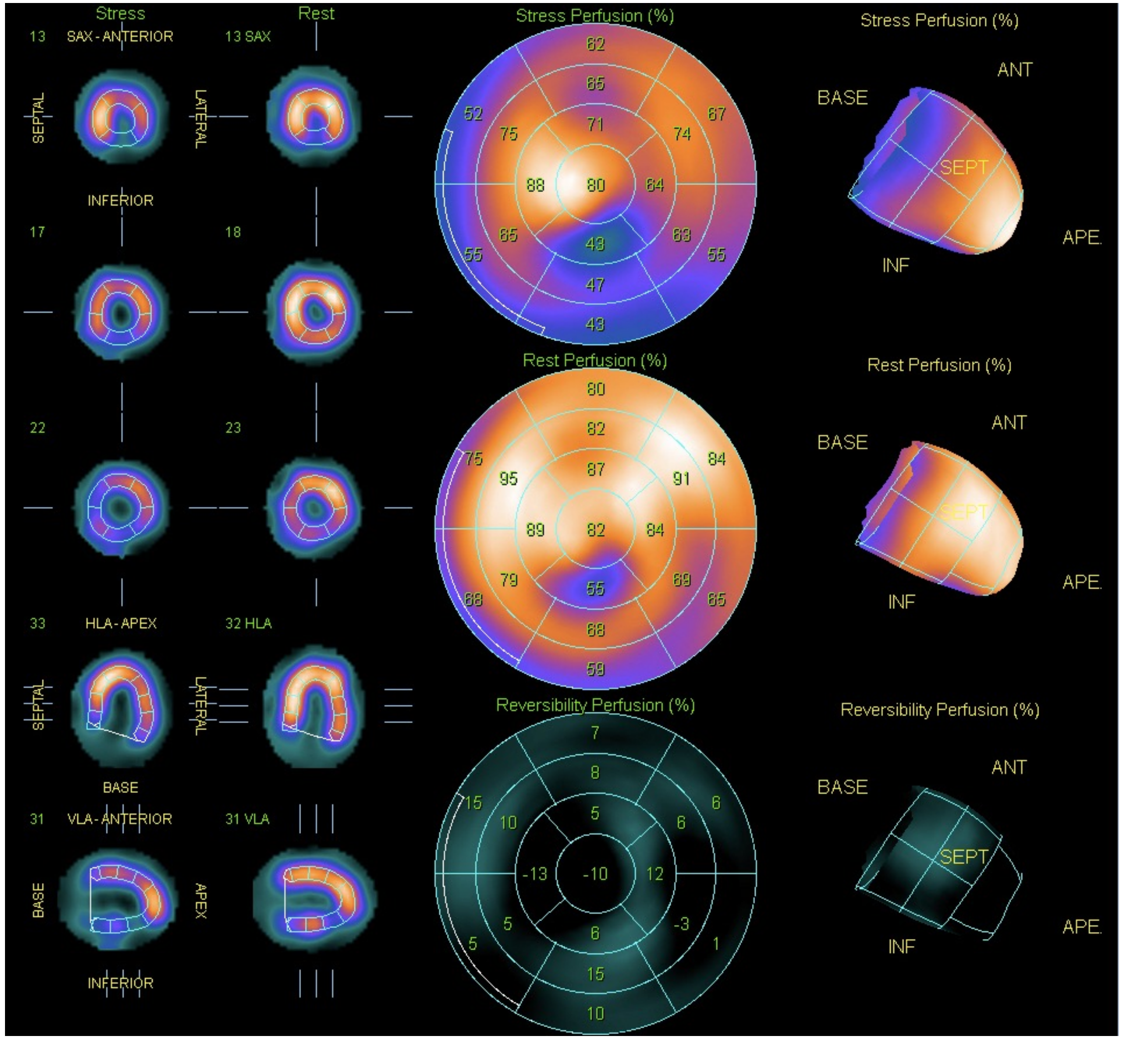

After LIPUS Therapy

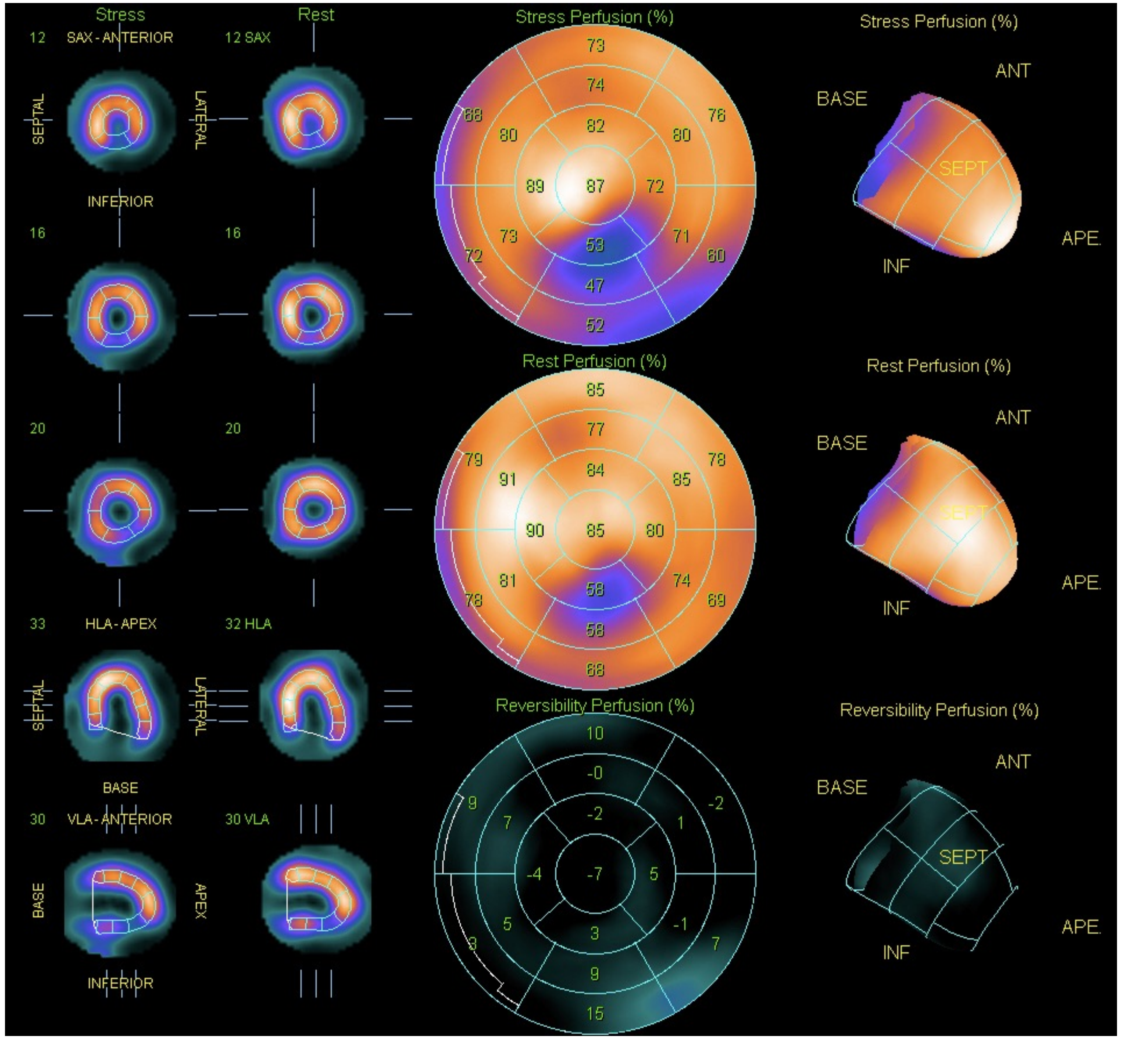

Supplement: S2 Fig — Correlation between the extent of baseline myocardial ischemia and that of post-treatment changes in myocardial ischemia evaluated by stress myocardial perfusion imaging. LIPUS: low-intensity pulsed ultrasound. (PDF) [file pone.0287714.s004.pdf]
