# Supplementary material for: A randomized, double-blind, placebo-controlled pilot trial of low-intensity pulsed ultrasound therapy for refractory angina pectoris
Source: PLoS One. 2023 Jun 23;18(6):e0287714. doi: 10.1371/journal.pone.0287714 (PMC10289346; doi:10.1371/journal.pone.0287714)
Supplement: S1 File — (PDF) [file pone.0287714.s005.pdf]

(医) 書式5

整理番号	平成25年度第5回IRB No. 133004
区分	■治験<br>□医薬品　■医療機器

西暦2013年08月26日

治験審査結果通知書

東北大学病院長　殿

東北大学病院治験審査委員会
宮城県仙台市青葉区星陵町1-1
海野　倫明

審査依頼のあった件についての審査結果を下記のとおり通知いたします。

記

被験薬の化学名<br>又は識別記号	T-75	治験実施計画書番号	C-02
治験課題名	虚血性心疾患患者を対象とした超音波血管新生療法装置T-75による超音波照射時の有効性及び安全性を評価するための臨床試験-多施設共同プラセボ対照無作為化二重盲検比較試験－（医師主導治験）		
審査事項<br>（審査資料）	■治験の実施の適否　（治験依頼書（西暦2013年07月30日付書式3写））<br>□治験の継続の適否<br>□重篤な有害事象<br>（□重篤な有害事象に関する報告書（西暦　年　月　日付書式12写））<br>（□有害事象に関する報告書（西暦　年　月　日付書式13写））<br>（□重篤な有害事象及び不具合に関する報告書（西暦　年　月　日付書式14写））<br>（□有害事象及び不具合に関する報告書（西暦　年　月　日付書式15写））<br>□安全性情報等<br>（安全性情報等に関する報告書（西暦　年　月　日付書式16写））<br>（安全性情報等に関する報告書（西暦　年　月　日付書式16写））<br>□治験に関する変更<br>（治験に関する変更申請書（西暦　年　月　日付書式10写））<br>□緊急の危険を回避するための治験実施計画書からの逸脱<br>（緊急の危険を回避するための治験実施計画書からの逸脱に関する報告書（西暦　年　月　日付書式 8 写））<br>□継続審査<br>（治験実施状況報告書（西暦　年　月　日付書式11写））<br>□その他（　　　　　　）		
審査区分	■委員会審査（審　査　日：西暦2013年08月26日）<br>□迅速審査　（審査終了日：西暦　年　月　日）		
審査結果	■承認　□修正の上で承認　□却下　□既承認事項の取り消し　□保留		
「承認」以外の<br>場合の理由等			
備考			

西暦2013年08月26日

自ら治験を実施する者　下川　宏明　殿

依頼のあった治験に関する審査事項について上記のとおり決定しましたので通知いたします。

東北大学病院　病院長

注）（長≠責）：本書式は治験審査委員会が正本を 1 部作成し、実施医療機関の長に提出する。治験審査委員会の決定と実施医療機関の長の指示が同じである場合には実施医療機関の長はその写 2 部の書式下部に通知日及び実施医療機関の長欄を記載し、治験依頼者及び治験責任医師にそれぞれ 1 部を提出する。異なる場合には参考書式 1 を使用する。
（長＝責）：本書式は治験審査委員会が正本を 1 部作成し、実施医療機関の長に提出する。治験審査委員会の決定と実施医療機関の長の指示が同じである場合には実施医療機関の長はその写 1 部の書式下部に通知日及び実施医療機関の長欄を記載し、治験依頼者に提出する。異なる場合には参考書式 1 を使用する。なお、治験責任医師欄は“該当せず”と記載する。
