# Supplementary material for: A randomized, double-blind, placebo-controlled pilot trial of low-intensity pulsed ultrasound therapy for refractory angina pectoris
Source: PLoS One. 2023 Jun 23;18(6):e0287714. doi: 10.1371/journal.pone.0287714 (PMC10289346; doi:10.1371/journal.pone.0287714)
Supplement: S2 File — (DOCX) [file pone.0287714.s006.docx]

Institutional Review Board of Tohoku University Hospital

1-1 Seiryo-machi

Aoba-ku, Sendai

980-8574 JAPAN

TEL: +81-22-717-7153

FAX: +81-22-717-7156

**Certificate of Approval**

Your new project entitled "A Randomized, Double-blind, Placebo-controlled Pilot Trial of Low-intensity Pulsed Ultrasound Therapy for Refractory Angina Pectoris" was reviewed by the Institutional Review Board of Tohoku University Hospital. And, this protocol has been approved on August 26, 2013.

Principal investigator: Hiroaki Shimokawa

Sincerely,

Tohoku University Hospital Director
