# Supplementary material for: A randomized, double-blind, placebo-controlled pilot trial of low-intensity pulsed ultrasound therapy for refractory angina pectoris
Source: PLoS One. 2023 Jun 23;18(6):e0287714. doi: 10.1371/journal.pone.0287714 (PMC10289346; doi:10.1371/journal.pone.0287714)
Supplement: S3 File — (PDF) [file pone.0287714.s007.pdf]

虚血性心疾患患者を対象とした超音波血管新生療法装置 T-75 による超音波照射時の有効性及び安全性を評価するための臨床試験

（医師主導治験）

治験実施計画書

治験調整医師

東北大学病院 循環器内科

下川 宏明

治験実施計画書番号:C-02

版番号:第 6.0 版

作成年月日:2020 年 2 月 26 日

本治験実施計画書は、本治験関係者に限定して提供される秘密文書として取り扱う。
被験者に説明する場合を除き、「自ら治験を実施する者」の文書による同意なしに第三者に開示することはできません。

目次

頁

1. 治験計画の経緯及び背景 1
1.1 治験実施計画の背景 1
1.2 治験機器開発の経緯 1
1.3 当該治験の位置づけ・妥当性 2
1.4 治験機器の説明 2
1.4.1 治験機器の名称及び構成 2
1.4.2 表示 4
1.4.3 保管及び管理手順 4
1.5 非臨床試験の要約 5
1.6 被験者に対する既知及び可能性のある危険（リスク）と利益（ベネフィット）の要約 5
1.6.1 治験機器に予想される危険 5
1.6.2 治験機器に予想される利益 5
2. 治験の目的 6
3. 治験のデザイン 6
3.1 評価項目 6
3.2 治験の種類及びデザイン 6
3.2.1 治験の種類 6
3.2.2 治験のデザイン 6
3.3 被験者の治験期間 7
3.4 治験実施予定期間 8
4. 被験者の選択・除外、中止基準 8
4.1 選択基準 8
4.2 除外基準 9
4.3 中止基準 10
5. 前治療及び併用療法 11
5.1 併用機器 11
5.2 併用薬 11
5.3 併用療法 11
6. 治験の手順 11
6.1 同意取得 11
6.2 スクリーニング 11
6.3 適応評価委員会による選択基準(4)の適否判断 12
6.4 被験者登録 12
6.4.1 盲検性の保持の手順 15
6.4.2 キーオープン 15
6.5 治験機器による治療 16

6.5.1 治療手順 16
6.5.2 治験機器使用手順 16
6.5.3 治験機器の使用上の注意 17
6.6 観察・検査スケジュール 17
6.6.1 各観察・検査ポイントの規定日及び許容範囲 21
6.6.2 調査・検査内容 22
6.6.3 中止後の検査・観察 29
7. 安全性評価 30
7.1 臨床検査値 30
7.2 有害事象 30
7.2.1 有害事象の定義 30
7.2.2 有害事象の記載 31
7.2.3 重篤な有害事象 31
7.2.4 有害事象の重篤性の判定 31
7.2.5 有害事象の治療のために取られた処置 31
7.2.6 治験機器に対して取られた処置 32
7.2.7 有害事象の転帰 32
7.3 不具合 33
7.3.1 不具合情報の記録 33
7.4 有害事象及び不具合発生時の対応 33
7.5 重篤な有害事象及び重篤な有害事象につながる恐れがある不具合が発生した場合の報告 33
7.6 新たな情報の提供 34
8. 有効性評価 34
8.1 主要評価項目 34
8.2 副次的評価項目 35
9. 統計解析 35
9.1 解析対象集団 35
9.2 有効性の解析 36
9.3 安全性の解析 36
9.4 症例数の設定 36
9.5 その他統計解析に関する事項 37
10. 治験の品質管理及び品質保証 37
10.1 原資料等の直接閲覧 37
10.2 モニタリング 37
10.3 監査 38
10.4 モニタリング及び監査への協力 38
10.5 治験責任医師保管書類 38
11. 倫理及び GCP 遵守 38

11.1 治験の倫理的実施 38
11.2 被験者への説明と同意の取得 38
11.3 同意取得の方法 39
11.4 同意説明文書の改訂 40
11.5 治験審査委員会(IRB) 40
11.6 被験者の人権保護に関する事項 40
11.7 データの二次利用について 40
12. 症例報告書 41
12.1 症例報告書の様式・構成 41
12.2 記入方法 41
12.3 症例報告書の提出 42
12.4 原資料 42
12.5 症例報告書中の記載内容を原資料とすべき項目の特定 42
13. 治験の費用負担及び補償 43
13.2 健康被害補償及び保険 43
13.3 予定される治験費用及び被験者への支払い 43
14. 治験実施計画書の逸脱又は変更及び改訂 44
14.1 治験実施計画書の逸脱又は変更 44
14.2 治験実施計画書の改訂 44
15. 治験の中止・中断・終了 44
15.1 治験の中止・中断 44
15.2 治験の終了 45
16. 効果安全性評価委員会について 45
17. 中央判定委員会について 45
18. 記録等の保存 45
19. 公表に関する取決め 46
20. 治験実施体制 46
21. 参考文献 46

略号及び用語の定義一覧表

略　号	内　容
BMI	Body Mass Index ボディマス指数
CABG	Coronary Artery Bypass Grafting 冠動脈バイパス術
CCS	Canadian Cardiovascular Society カナダ心臓血管協会
CRC	Clinical Research Coordinator 治験コーディネーター
CRF	Case Report Form 症例報告書
CRO	Contract Research Organization 医薬品開発業務受託機関
CVIT	Japanese Association of Cardiovascular Intervention and Therapeutics<br>日本心血管インターベンション治療学会
GCP	Good Clinical Practice 医薬品の臨床試験の実施の基準に関する省令
HCG	Human Chorionic Gonadotropin ヒト絨毛性ゴナドトロピン
IRB	Institutional Review Board 治験審査委員会
MACE	Major Adverse Cardiac Events 主要心血管イベント
MRI	Magnetic Resonance Imaging 核磁気共鳴画像法
PCI	Percutaneous Coronary Intervention 経皮的冠動脈インターベンション
QOL	Quality Of Life 生活の質
SSS	Summed Stress Score 負荷時の欠損スコア（負荷時の虚血や血流低下を反映）
SRS	Summed Rest Score 安静時の欠損スコア（心筋梗塞や線維化の量に相当）
SDS	Summed Difference Score SSS と SRS の差（負荷により誘発される虚血に相当）

要約

治験名	虚血性心疾患患者を対象とした超音波血管新生療法装置 T-75 による超音波照射時の有効性及び安全性を評価するための臨床試験　（医師主導治験）
目　的	虚血性心疾患に対する新しい治療法を開発することにより、患者の生活の質（QOL：Quality Of Life）を改善すること
治験の種類	医療機器
治験方法	多施設共同プラセボ対照無作為化被験者・評価者盲検比較試験
対象患者	虚血性心疾患（薬物療法に対して抵抗性を有し、かつ、経皮的冠動脈インターベンション又は冠動脈バイパス術による治療が困難な狭心症症例）
選択基準	(1) 文書で本人より治験参加の同意が得られている患者<br>(2) 同意取得時に年齢が 20 歳以上の患者<br>(3) 治験治療ならびに実治療の初回予定日から 6 日間の入院が可能な患者<br>[登録時の確認事項]<br>(4) 登録時に虚血性心疾患の薬物療法に対して抵抗性を有し、かつ経皮的冠動脈インターベンション又は冠動脈バイパス術による治療が困難、あるいは利益に比してリスクが高い虚血性心疾患と診断された患者<br>適否の判断は、①日本心臓血管外科学会専門医及び②日本心血管インターベンション治療学会専門医のそれぞれ 1 名からなる適応評価委員会（「別紙 1」参照）が行う。<br>(5) 登録時にガイドラインに沿った十分な薬物治療下にもかかわらず、平均して 1 週間に 1 回以上、狭心症発作で硝酸薬を服用あるいは吸入する患者<br>(6) 登録時に負荷心筋シンチグラフィーで心筋虚血が示唆される領域が存在する患者<br>(7) 治験ノートの記載が可能な患者（同意取得翌日から登録前日までの 28 日間以上で、記載率が 70%以上）
除外基準	(1) 登録時に、心臓超音波装置にて目標範囲を同定できないと判断した患者<br>(2) 登録時に、心臓超音波検査の画像診断で心内血栓を認めると判断した患者<br>(3) 登録時に、最後の経皮的冠動脈インターベンション・冠動脈バイパス術から 28 日以内の患者<br>(4) 登録時から前 84 日以内に、Q 波心筋梗塞をおこしている患者<br>(5) 登録時から前 42 日以内に、非 Q 波心筋梗塞をおこしている患者<br>(6) 登録時に、心原性ショックあるいは心不全増悪状態の患者（強心薬・血管拡張薬など心血管作動薬の持続静注を要する患者）<br>(7) 同意取得時から前 3 年以内のうち直近の心臓カテーテル検査後に、狭心症パターンや臨床状態の変化した患者<br>(8) 登録時に、コントロールのできていない糖尿病性網膜症（活動性の眼底出血を認める）の患者<br>(9) 登録時に、悪性腫瘍が併存する、あるいは登録時から 5 年以内に悪性腫瘍の手術を受けている患者

	(10) 妊娠している患者及び妊娠の可能性のある患者<br>(11) 他の臨床試験や治験に参加している患者<br>(12) その他、治験責任医師又は治験分担医師が不適格と判断した患者
治験機器	(株) 日立製作所　超音波血管新生療法装置 T-75
治験期間	同意取得日から最終観察日（実治療観察期検査 5）まで
検査・観察項目	別表のとおり
有効性評価項目	(1) 主要評価項目<br>・硝酸薬の週平均使用頻度<br>(2) 副次的評価項目<br>・自覚症状（CCS クラススコア、胸痛の週平均発現頻度）<br>・運動耐用能（トレッドミル運動負荷心電図、6 分間歩行試験）<br>・心筋血流（アデノシンを用いた薬物負荷心筋シンチグラフィー）<br>・左室駆出率 40%以下の低心機能例における心機能（心臓超音波検査、心臓 MRI で評価）
安全性評価項目	(1) 臨床検査値<br>(2) 胸部 X 線<br>(3) 実治療初回実施後 1 年までの主要心血管イベント：MACE（心血管死、非致死性心筋梗塞、入院を要する不安定狭心症・心不全）の発生頻度<br>(4) 有害事象<br>(5) 不具合
統計解析	有効性の評価は、平均値、中央値、及び、改善した被験者の割合を、プラセボ治療群と実治療群との間で比較することにより行う。<br>安全性については、実治療初回実施後1年までの MACE 発生頻度が 40%を越えないことを確認することにより評価を行う。
目標登録症例数	80 例（各群 40 例）
中止基準	(1) 症状悪化（原疾患の悪化）のため、治験の継続が困難となった場合<br>(2) 合併症の増悪のために治験の継続が困難な場合<br>(3) 治験の継続が不可能な有害事象が発現した場合<br>(4) 被験者が死亡した場合<br>(5) 治験実施計画書からの重大な逸脱が明らかになった場合<br>(6) 被験者が来院しない場合<br>(7) 妊娠が判明した場合<br>(8) 被験者より治験参加同意の撤回があった場合<br>(9) 被験者登録後に適格性を満たしていないことが判明した場合<br>(10) 治験全体が中止された場合<br>(11) その他、治験責任医師又は治験分担医師が治験を中止すべきであると判断した場合
実施医療機関	別紙 1 参照
使用方法	被検者を仰臥位とし、超音波プローブを前胸壁に当てて心臓を観察し、虚血部位を描写する。この時点で治療用の超音波発生条件に設定を変更することにより、描写範囲が治

	療範囲となる。虚血部位の同定は、複数の画像診断の結果をもとに行う。一断面につき20分±1分照射を行い、5分以上間隔をあけ、心基部から心尖部の計3断面照射する。治療時の超音波の設定条件は、培養細胞と動物実験による基礎データをもとに決定した（0.25 W/cm^2, 1.875MHz, 矩形32サイクル)。
治験実施期間	2013年11月～2023年3月

観察・検査スケジュール

		同意取得時	スクリーニング時[5]	登録時	治験治療												
					治験治療							観察期					
					治験治療直前	1回目		2回目		3回目		14日目	検査1	検査2	70日目	検査3[6]	検査4[9]
規定日		−86〜−30日	−86〜−2日	−14〜−2日	0日		1日	2日	3日	4日	5日	14日	28日	56日	70日	84日	1年
許容範囲（日）					起点日		+3	+6	+9	+12	+14	±3	+7	±14	±3	±14	±28
同意取得		○															
登録				○													
超音波治療						○		○		○							
治験機器の調査[8]						○		○		○							
被験者背景			○														
体重・BMI[16]			○			○	○	○	○	○	○		△	△		○	○
血圧測定[16]			○			○[4]	○	○[4]	○	○[4]	○		△	△		○	○
体温測定						○[4]	○	○[4]	○	○[4]	○						
妊娠検査			○													○	
身体所見[16]			○			○	○	○	○	○	○		△	△		○	○
硝酸薬の使用記録[3]			←→			←										→	○
胸痛の発現記録[3]			←→			←										→	○
有害事象、主要心血管イベント[10]						←										→	○
CRCによる電話等での聴取[16]												○	△	△	○		
CCSｸﾗｽｽｺｱ			○													○	
併用薬、併用療法調査			○	←												→	○
臨床検査[12]	血液検査[18]		○				○		○		○					○	
	血液生化学検査[18]		○				○		○		○					○	
	血液特殊検査[14]		○								○					○	
	安静時12誘導心電図		○				○		○		○					○	
	ﾄﾚｯﾄﾞﾐﾙ運動負荷心電図[17]	○														○	
	6分間歩行試験[15・17]	△														△	
画像検査[12]	胸部X線		○				○		○		○					○	
	心臓超音波検査		○													○	
	心臓MRI[13]	△														△	
	負荷心筋ｼﾝﾁｸﾞﾗﾌｨｰ[1]	○														○	
	心臓ｶﾃｰﾃﾙ検査[2]	○															

	実治療													中止時[11]
	実治療							観察期						
	実治療前日[6・7]	1回目		2回目		3回目		14日目	検査1	検査2	70日目	検査3	検査5	
規定日	−1日	0日	1日	2日	3日	4日	5日	14日	28日	56日	70日	84日	1年	
許容範囲（日）[6・7]	−1	起点日	+3	+6	+9	+12	+14	±3	+7	±14	±3	±14	±28	
同意取得														
登録														
超音波治療		○		○		○								
治験機器の調査[8]		○		○		○								
被験者背景														
体重・BMI[16]	△	○	○	○	○	○	○		△	△		○	○	○
血圧測定[16]	△	○[4]	○	○[4]	○	○[4]	○		△	△		○	○	○
体温測定		○[4]	○	○[4]	○	○[4]	○							
妊娠検査														
身体所見[16]	△	○	○	○	○	○	○		△	△		○	○	○
硝酸薬の使用記録[3]	←												→	○
胸痛の発現記録[3]	←												→	○
有害事象、主要心血管イベント[10]	←												→	○
CRCによる電話等での聴取[16]								○	△	△	○			
CCS ｸﾗｽｽｺｱ												○		○
併用薬、併用療法調査	←												→	○
臨床検査[12] 血液検査			○		○		○					○		○
臨床検査[12] 血液生化学検査			○		○		○					○		○
臨床検査[12] 血液特殊検査[14]							○							
臨床検査[12] 安静時12誘導心電図			○		○		○					○		○
臨床検査[12] ﾄﾚｯﾄﾞﾐﾙ運動負荷心電図												○		
臨床検査[12] 6分間歩行試験[15]												△		
画像検査[12] 胸部X線			○		○		○					○		○
画像検査[12] 心臓超音波検査												○		○
画像検査[12] 心臓MRI[13]												△		
画像検査[12] 負荷心筋ｼﾝﾁｸﾞﾗﾌｨｰ[15]												○		
画像検査[12] 心臓ｶﾃｰﾃﾙ検査														

○：必須とする検査項目。

△：可能な限り実施する検査項目。

1 症状に変化がない場合、同意取得日から 84 日前までの結果を使用可能とする。実治療観察期検査 3 は必須としない。

2 症状に変化がない場合、同意取得日から 3 年前までの結果を使用可能とする。また、合併症などのため施行困難な場合は、他の検査所見を参考に判断する。

3 同意取得日から治験ノート記載を開始し、記録記載が可能な患者だけを登録する。

4 治療の前後で測定する。

5 スクリーニング時検査のトレッドミル運動負荷心電図、6 分間歩行試験、心臓 MRI については、治験治療初回実施前までに実施すればよい。

6 観察期検査 3（治験治療初回実施後 84 日）終了後 28 日以内に実治療初回を実施する。

7 実治療前日は必須としない。実施しない場合は、実治療実施前に当該検査を実施し、実治療実施の可否を評価する。また、治験治療から実治療を継続し、観察期検査 3 と実治療前日が同日の場合、観察期検査 3 の検査・観察項目を実施する。

8 治験機器の調査は、6.6.2 調査・検査内容(21)を実施する。

9 治験治療検査 4 は、実治療に移行しなかった被験者に行う。

10 主要心血管イベント：MACE（心血管死、非致死性心筋梗塞、入院を要する不安定狭心症・心不全）

11 治験中止時は、可能な限り規定された検査・観察を実施する。

12 同意取得から実治療観察期検査 3 まで及び中止時に行う臨床検査及び心臓カテーテル検査以外の画像検査は、原則同一の医療機関で実施する。

13 撮像可能な場合、同意取得日から 84 日前までの結果を使用可能とする。心臓 MRI 撮像は必須としない。

14 血液特殊検査は、血漿を冷凍保管し、中央検査を実施する。なお、やむを得ない事情により、各治療 3 回目翌日の実施が困難な場合は、各治療 3 回目終了後同日にも実施可能とする。

15 6 分間歩行試験、実治療観察期検査 3 の負荷心筋シンチグラフィーは必須としない。

16 観察期検査 1、観察期検査 2 は必須としない。CRC による電話等での聴取は、観察期検査 1、観察期検査 2 に受診できない被験者に対して行う。

17 症状に変化がない場合、同意取得日から 1 年前までの結果を使用可能とする。

18 スクリーニング時の血液検査及び血液生化学検査については、症状に変化がない場合、同意取得日から 84 日前までの結果を使用可能とする。

1. 治験計画の経緯及び背景

1.1 治験実施計画の背景

虚血性心疾患に対する標準的治療は、生活習慣の改善を基本に、①薬物療法、②経皮的冠動脈インターベンション（Percutaneous Coronary Intervention: PCI）、③冠動脈バイパス術（Coronary Artery Bypass Grafting: CABG）の 3 本柱から成る。しかし、近年我が国では、人口の高齢化や食生活の欧米化に伴い、これら従来の治療法では十分な効果が得られない重症例（例えば、びまん性狭窄病変を持つ症例や慢性期にバイパスグラフトが閉塞した例）が増加してきている。このような症例では、胸痛や息切れのため生活の質が大きく低下しており、医学的にも社会的にも問題となっている。

下川らは、低出力の衝撃波を用いた非侵襲的な血管新生療法（『低出力体外衝撃波治療法』）を開発し、基礎研究の結果をもとに、狭心症患者を対象に臨床試験を行い、有効性・安全性を確認した（参考文献 1～4）。現在では、狭心症に対する低出力体外衝撃波治療は、欧州を中心に世界 10 ヵ国以上で行われており、既に 3000 人以上の患者が治療を受けている。

一方、近年、超音波照射による血管新生の論文報告がなされている（参考文献 5～7）。超音波は、衝撃波を心臓病治療に用いる際に懸念されるような肺傷害の危険性がなく扱いやすい。また、超音波画像診断装置は世界的に幅広く普及していることから、もし超音波画像診断装置の超音波発生条件の設定を変更することにより、超音波画像診断装置を治療装置へ応用できれば、インパクトは非常に大きく、普及が期待できると考えた。そこで、下川らは基礎研究を行い、培養細胞を用いた検討において、血管新生を促進する超音波発生条件を明らかにした。また、ブタ慢性心筋虚血モデルを用いた検討において、超音波照射による血管新生を確認した。これらの結果をもとに、狭心症患者を対象とした医師主導治験を行い、治療機器としての有効性・安全性を検討したいと考えている。

1.2 治験機器開発の経緯

下川らは、低出力の衝撃波を用いた非侵襲的な血管新生療法（『低出力体外衝撃波治療法』）を開発してきた。これは、尿路結石破砕治療に用いられる出力の約 10 分の 1 という弱い出力の衝撃波を体外から虚血心筋に照射すると、心筋における血管増殖因子の発現が亢進し、血管新生が促進されて心筋虚血が改善されるというもので、これまでにブタの慢性心筋虚血モデルを用いた基礎研究や臨床試験でその有効性・安全性が報告されている（参考文献 1～4）。なお、『低出力体外衝撃波治療法』については重症虚血性心疾患患者（狭心症）に対する治療法として、平成 22 年度に厚生労働省より高度医療（第 3 項先進医療）に承認され、現在、東北大学病院ならびに石川県立中央病院において治療が行われている。

一方、近年、低出力衝撃波と同様に、超音波照射により、血管新生が誘導されるという報告がなされている（参考文献 5～7）。下川らは、超音波を照射することにより、培養血管内皮細胞において、血管増殖因子の産生が亢進することを確認し、さらに、ブタの慢性心筋虚血モデルにおいて、血管新生が促進されて心筋虚血が改善し、心機能が改善するこ

とを確認した（参考文献 8)。また、ブタの慢性心筋虚血モデルにおいて、超音波治療法による心機能改善効果は、低出力体外衝撃波治療法とほぼ同等であった（参考文献 8)。これらのことから、超音波診断装置を治療用として臨床応用するのに必要な準備が整ったと判断し、計画を立案した。

1.3 当該治験の位置づけ･妥当性

ブタ慢性心筋虚血モデルにおいて、超音波治療は、低出力体外衝撃波治療と同様の治療効果をもたらすことが確認された。超音波治療法は、低出力体外衝撃波治療法と比べて、共通の 1 つのプローブで診断と治療を同時に行えるとともに、肺損傷の可能性がないというメリットがある。また、超音波診断装置は、画像診断装置として、世界的にもクリニックから大病院まで幅広く普及している。今回申請する超音波治療法では、既存のハードウェア（超音波診断装置）の超音波照射条件をソフトウェア対応で変更することにより、治療装置へ応用することを可能にすることから、今後の普及が期待される。

当該治験は人での POC（Proof Of Concept）取得とともにその安全性を確認することを主眼に実施するものであり、有効性及び安全性が確認された場合に当該治験の成功とみなし、当該治験成績をもって、承認申請を行う予定である。

1.4 治験機器の説明

当該治験機器は、超音波診断装置の機能を利用して虚血している心筋部位に診断レベルの超音波を照射し、血管新生療法を行う装置である。

1.4.1 治験機器の名称及び構成

当該治験機器は、診断装置本体、観測用モニタ部及びプローブホルダーなどの付属品を総称して「T-75」という。ただし、汎用品はこれらに含まれない。

［本体］

(①－1 プローブコネクタ部、 ①－2 ケーブルハンガー、 ①－3 ケーブルフック、
①－4 外部記録装置 搭載台、 ①－5 電源入力ケーブル、 ①－6 ハンドル、
①－7 モニタアーム、 ①－8 モニタホームポジションロックレバー、
①－9 操作パネル昇降ペダル、 ①－10 キャスタ、 ①－11 キャスタロック・リリースペダル、 ①－12 操作パネルスライド・旋回レバー、 ①－13 操作パネルホームポジションロックレバー、 ①－14 フットスイッチ接続接栓)

［観測用モニタ部］（汎用機器を使用）

②－1 モニタ用ハンドル

［付属品］

③－1 プローブホルダ

［オプション］

④－1 生体信号ユニット、 ④－2 アナログビデオ信号出力ユニット（プリント基板)、④－3 DV 出力ユニット（プリント基板)、 ④－4 フルキーボードユニット、 ④－5 フットスイッチ、 ④－6 外部記録装置（白黒プリンタ、カラープリンタ、DVD レコーダー、ドライブベイユニット)、 ④－7 インディペンデントプローブ接続ユニット、④－8 ワイヤレスリモートコントローラー、 ④－9 プローブホルダ

［操作パネル］

⑤－1 USB 接栓、 ⑤－2 パネル用ハンドル、 ⑤－3 赤外線受光部

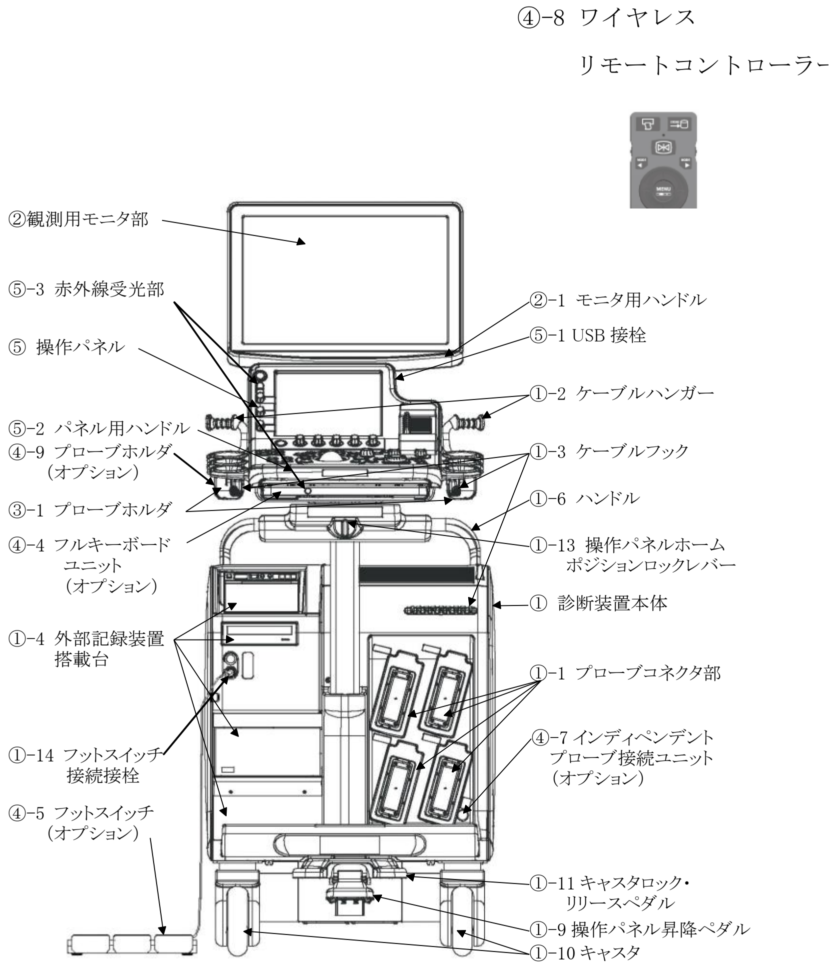

図 1-1 システム構成図

1.4.2 表示

T-75　　治験用

＜注意＞使用時には治験機器管理表に記録すること

治験調整医師
東北大学病院　循環器内科　教授　下川宏明
宮城県仙台市青葉区星陵町 1-1

シリアルナンバー(S/N)　：

使　用　期　限　：	年	月

上記のラベルは、下図の位置に貼付する。

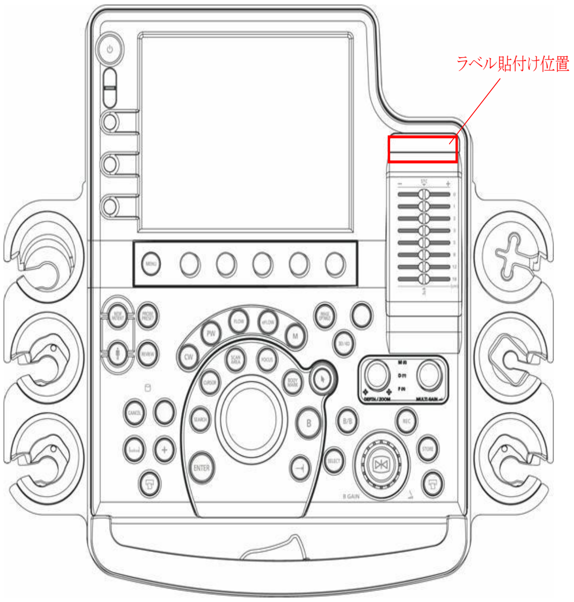

1.4.3 保管及び管理手順

当該治験機器は各治験実施医療機関にて保管する。治験機器の適切な保管及び管理のため、治験機器管理者を置くものとする。治験機器管理者は「治験機器の管理に関する手順書」に準じ、治験機器を適切に保管するとともに、治験機器の使用状況及び治験の進行状況を把握するために治験機器管理表を作成する。

1.5 非臨床試験の要約

診断に用いられる強度の超音波を、ある特定の照射設定でブタ虚血心筋に照射すると、以下の結果が得られた。

・血管内皮増殖因子の発現が亢進

・毛細血管密度の増加が促進

・心筋血流が改善

・心機能が改善

さらに、副作用や合併症は認められなかった。治療時の超音波の設定条件は、培養細胞と動物実験による基礎データをもとに決定した（0.25 W/cm^2, 1.875MHz, 矩形 32 サイクル)。

1.6 被験者に対する既知及び可能性のある危険（リスク）と利益（ベネフィット）の要約

1.6.1 治験機器に予想される危険

超音波診断に関する有害事象として同添付文書に挙げられている既知の事象、さらに当該治験機器の虚血性心疾患適応において起こり得る有害事象等を機器概要書「リスク解析表（43～44 ページ)」から抜粋した。

・プローブ表面温度上昇による低温やけど

・治療中に照射部位がずれ、治療目的以外の部位に照射される。

・プローブ固定用ベルトを強く締めすぎ、息苦しくなる。

・プローブを押し当てる力が強すぎて痛い。

・プローブを押し当てる力が弱すぎて治療用超音波が充分患部に届かない。

1.6.2 治験機器に予想される利益

当該治験機器は、広く普及し安全性が確認されている超音波診断装置の機能を利用しているので、身近な施設で手軽に治療を受けることができるようになると考える。

2. 治験の目的

虚血性心疾患に対する新しい治療法を開発することにより、患者の生活の質（Quality Of Life:QOL）を改善することを目標とする。具体的には、虚血性心疾患患者を対象に超音波治療を行い、硝酸薬の週平均使用頻度（主要評価項目)、自覚症状・運動耐用能・心筋血流・心機能（副次評価項目）の改善、及び主要心血管イベント（安全性）について評価する。

3. 治験のデザイン

3.1 評価項目

(1) 有効性評価項目

1)主要評価項目

・硝酸薬の週平均使用頻度

2) 副次的評価項目

・自覚症状（CCS クラススコア、胸痛の週平均発現頻度)

・運動耐用能（トレッドミル運動負荷心電図、6 分間歩行試験)

・心筋血流（アデノシンを用いた薬物負荷心筋シンチグラフィー)

・左室駆出率 40%以下の低心機能例における心機能(心臓超音波検査、心臓 MRI で評価)

(2) 安全性評価項目

1) 臨床検査値

2) 胸部 X 線

3) 実治療初回実施後 1 年までの主要心血管イベント：MACE（心血管死、非致死性心筋梗塞、入院を要する不安定狭心症・心不全）の発生頻度

4) 有害事象

5) 不具合

＜設定根拠＞

当該治験は、日本人虚血性心疾患患者で初めて実施する試験であることから、安全性及び忍容性、ならびに有効性を確認するために設定した。

3.2 治験の種類及びデザイン

3.2.1 治験の種類

多施設共同プラセボ対照無作為化被験者・評価者盲検比較試験

3.2.2 治験のデザイン

選択基準に合致し、除外基準に抵触しない虚血性心疾患患者を被験者として、無作為に実治療群とプラセボ治療群に振り分ける。治療には、超音波発生条件の設定を変更できる心臓超音波装置（日立製作所製）を用いる。治験治療は入院下で症例登録の翌日以降に開

始し、1 日 3 回を 1 セットとし、隔日に合計 3 セットを実施する。症例登録から治験治療初回実施後 84 日間は、内服薬及び外用薬による薬物療法を原則変更しない。

治験治療初回実施後 84 日検査を実施後、28 日以内に実治療を開始する。治験治療と同様に、入院下で 1 日 3 回を 1 セットとし、隔日に合計 3 セットを行い、実治療初回実施後 84 日検査で安全性・有効性を評価し、実治療初回実施後 1 年検査で安全性を評価する。

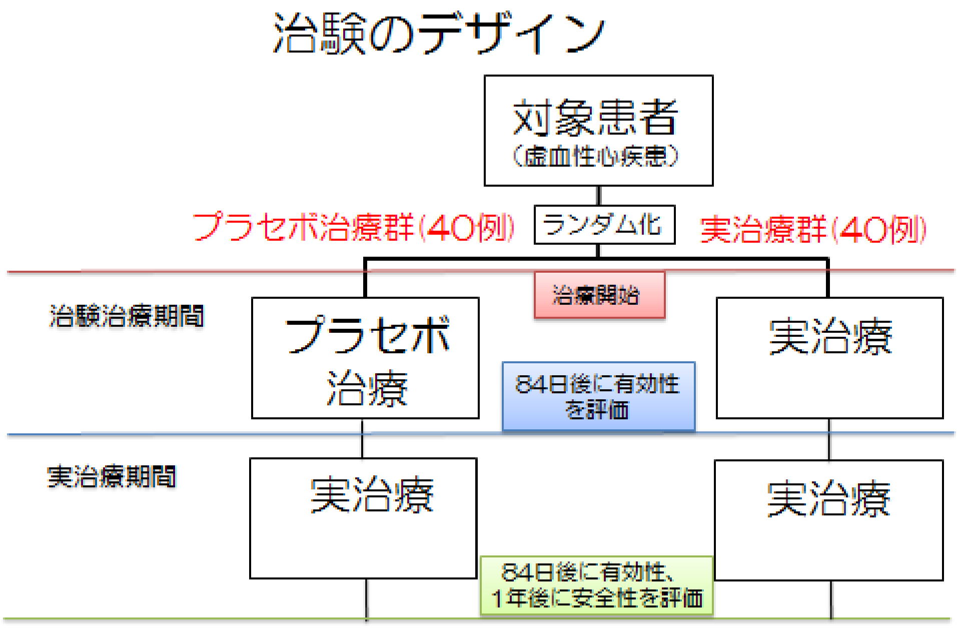

＜設定根拠＞

動物実験において、治療効果は治療開始 1 か月後までには認められている。日常生活における症状などに対する治療効果を判定するには、最後の治療から評価までの期間を 1～2 か月空ける必要がある。一方、フォローアップ期間が長期（例えば 6 か月以上）になると、（もし本治療法が有効であった場合）プラセボ治療を受けた被験者が相対的に不利益を受ける可能性があると考えられる。以上から、フォローアップ期間は 3 か月が適当と判断した。

3.3 被験者の治験期間

各被験者の治験期間は、同意取得日から最終観察日（実治療観察期検査 5）までとする。

＜設定根拠＞

治療の有効性は、治験治療初回実施後 84 日に評価を行う。続いて、プラセボ群に振り分けられた患者に対する倫理的な配慮から両群に実治療を実施し、同様に実治療初回実施後 84 日に有効性ならびに安全性評価を行う。さらに、主要心血管イベント：MACE（心血管死、非致死性心筋梗塞、入院を要する不安定狭心症・心不全）の評価を行うには 3 か月間では発生率が低く不十分と考えられるため、実治療初回実施後 1 年に検査等を行い、他の論文との間接比較することにより、治療の安全性を評価する。

3.4 治験実施予定期間

2013 年 11 月～2023 年 3 月

4. 被験者の選択・除外、中止基準

治験参加の同意が本人から文書で得られ、登録時に「4.1　選択基準」すべてを満たし、「4.2 除外基準」のいずれにも抵触しない被験者を当該治験の対象とする。登録後、治験治療実施までにおいても同基準を準拠する。

4.1 選択基準

(1) 文書で本人より治験参加の同意が得られている患者

(2) 同意取得時に年齢が 20 歳以上の患者

(3) 治験治療ならびに実治療の初回予定日から 6 日間の入院が可能な患者

［登録時の確認事項］

(4) 登録時に虚血性心疾患の薬物療法に対して抵抗性を有し、かつ経皮的冠動脈インターベンション又は冠動脈バイパス術による治療が困難、あるいは利益に比してリスクが高い虚血性心疾患と診断された患者

適否の判断は、①日本心臓血管外科学会専門医及び②日本心血管インターベンション治療学会専門医のそれぞれ 1 名から成る適応評価委員会（「別紙 1」参照）が行う。適応評価委員会については別途手順書を定める。

(5) 登録時にガイドラインに沿った十分な薬物治療下にもかかわらず、平均して 1 週間に 1 回以上、狭心症発作で硝酸薬を服用あるいは吸入する患者

(6) 登録時に負荷心筋シンチグラフィーで心筋虚血が示唆される領域が存在する患者

(7) 治験ノートの記載が可能な患者（同意取得翌日から登録前日までの 28 日間以上で、記載率が 70%以上）

＜設定根拠＞

(1)、(2) 同意能力の備わった 20 歳以上の患者。

(3) 当該治験では入院を必須としたため設定した。

(4) 経皮的冠動脈インターベンションや冠動脈バイパス術の施行により、全身状態を悪化させることなく症状の改善が見込める患者については、それらの治療法を優先する。

(5) 標準的な薬物治療下でも狭心症発作を生じる狭心症患者のうち、経皮的冠動脈インターベンションや冠動脈バイパス術による治療が困難、あるいは、利益に比してリスクが高いと考えられる患者では、胸痛発作を避けるために日常生活が著しく制限されており、QOL が低下している。このような患者の多くは、胸痛発作なく活動性を上げることを望んでいるため、本治療法の対象として適当と考えられる。ただし、硝酸薬の使用頻度が少ない場合、治療効果の判定が困難となることから、その使用頻度が 1 週間に 1 回未満の患者は除外とする。

(6) 狭心症発作などの自覚症状に加えて、画像診断（負荷心筋シンチグラフィー、可能であれば心臓MRI）を用いて、客観的な血流評価も行う。
(7) 治験ノートの記載内容が主要評価項目なので、記載可能であることを必須とする。

「ガイドライン」：以下の1)，2)の診療ガイドラインを指す。
1) 日本循環器学会によるガイドライン http://www.j-circ.or.jp/
・心筋梗塞二次予防に関するガイドライン（2011年改訂版）：ホームページ公開のみ。
・慢性虚血性心疾患の診断と病態把握のための検査法の選択基準に関するガイドライン（2010年改訂版）：ホームページ公開のみ。
・慢性心不全治療ガイドライン（2010年改訂版）：ホームページ公開のみ。
・冠動脈疾患におけるインターベンション治療の適応ガイドライン（冠動脈バイパス術の適応を含む）
—待機的インターベンション—：*Circ J.* 2001;65 Sup VI 及び *Circ J.* 2000;64 Sup VI.
2) アメリカ心臓協会（American Heart Association）によるガイドライン
2007 chronic angina focused update of the ACC/AHA 2002 Guidelines for the management of patients with chronic stable angina: a report of the American College of Cardiology/American Heart Association Task Force on Practice Guidelines Writing Group to develop the focused update of the 2002 Guidelines for the management of patients with chronic stable angina. Fraker TD Jr, Fihn SD, Gibbons RJ, Abrams J, Chatterjee K, Daley J, Deedwania PC, Douglas JS, Ferguson TB Jr, Fihn SD, Fraker TD Jr, Gardin JM, O'Rourke RA, Williams SV, Smith SC Jr, Jacobs AK, Adams CD, Anderson JL, Buller CE, Creager MA, Ettinger SM, Halperin JL, Hunt SA, Krumholz HM, Kushner FG, Lytle BW, Nishimura R, Page RL, Riegel B, Tarkington LG, Yancy CW; American College of Cardiology; American Heart Association; American College of Cardiology/American Heart Association Task Force on Practice Guidelines Writing Group. *Circulation*. 2007;116:2762-72.

4.2 除外基準
(1) 登録時に、心臓超音波装置にて目標範囲を同定できないと判断した患者
(2) 登録時に、心臓超音波検査の画像診断で心内血栓を認めると判断した患者
(3) 登録時に、最後の経皮的冠動脈インターベンション・冠動脈バイパス術から28日以内の患者
(4) 登録時から前84日以内に、Q波心筋梗塞をおこしている患者
(5) 登録時から前42日以内に、非Q波心筋梗塞をおこしている患者
(6) 登録時に、心原性ショックあるいは心不全増悪状態の患者（強心薬・血管拡張薬など心血管作動薬の持続静注を要する患者）

(7) 同意取得時から前 3 年以内のうち直近の心臓カテーテル検査後に、狭心症パターンや臨床状態の変化した患者

(8) 登録時に、コントロールのできていない糖尿病性網膜症（活動性の眼底出血を認める）の患者

(9) 登録時に、悪性腫瘍が併存する、あるいは登録時から 5 年以内に悪性腫瘍の手術を受けている患者

(10) 妊娠している患者及び妊娠の可能性のある患者

(11) 他の臨床試験や治験に参加している患者

(12) その他、治験責任医師又は治験分担医師が不適格と判断した患者

＜設定根拠＞

(1) 超音波治療は、照射目標部位の同定できない症例には照射不可能なため対象から除外する。

(2) 心内血栓を認める場合、血栓に与える影響（血栓遊離による塞栓症のリスク）が不明であるため除外する。

(3) 最後の経皮的冠動脈インターベンション・冠動脈バイパス術から 28 日以内の症例では、超音波治療前の自覚症状を十分に評価できないため除外とする。

(4)-(7) の症例は、不安定な病態が、治療効果の評価に影響を与える可能性があるため除外とする。

(8)、(9) 本治療法は、血管新生を促進することが有効性の主な機序と考えられているので、血管新生の促進により病態が増悪する可能性がある疾患を有する被験者を除外とする。

(10)-(12) 倫理的に被験者の安全性を担保するため。

4.3 中止基準

(1) 症状悪化（原疾患の悪化）のため、治験の継続が困難となった場合

(2) 合併症の増悪のために治験の継続が困難な場合

(3) 治験の継続が不可能な有害事象が発現した場合

(4) 被験者が死亡した場合

(5) 治験実施計画書からの重大な逸脱が明らかになった場合

(6) 被験者が来院しない場合

(7) 妊娠が判明した場合

(8) 被験者より治験参加同意の撤回があった場合

(9) 被験者登録後に適格性を満たしていないことが判明した場合

(10) 治験全体が中止された場合

(11) その他、治験責任医師又は治験分担医師が治験を中止すべきであると判断した場合

＜設定根拠＞

倫理的に患者の安全性を担保するため。

5. 前治療及び併用療法

5.1 併用機器

治験期間を通じて、当該治験機器以外の血管新生による虚血性心疾患の治療を目的とする医療機器の使用は禁止する。その他医療機器の使用は、体内埋め込み型医療機器の併用も含めて、特に制限しない。

5.2 併用薬

同意取得以前から使用中の治療薬は、併用可能とする。また、虚血性心疾患治療薬の用法・用量は、原則として変更しない。ただし、主治医が必要と判断した際には、病状に応じた調整はやむを得ない。

抗がん剤については、血管新生や組織再生を強力に抑制する恐れがあり、撹乱要因となるため併用禁止とする。

5.3 併用療法

治験期間を通じて、当該治験機器以外の血管新生による虚血性心疾患の治療を目的とした治療は禁止する（例：低出力体外衝撃波治療、骨格筋由来細胞シート心表面移植術等）。

6. 治験の手順

6.1 同意取得

(1) 治験責任医師又は治験分担医師は被験者の選定にあたり、被験者の健康状態、症状、年齢、性別、同意能力、他の治験への参加の有無等を考慮し、被験者を治験の対象とすることの適否を慎重に検討する。

(2) 治験責任医師又は治験分担医師は、当該治験の対象として適切と判断した被験者に対し、当該治験の説明を十分に行い、文書による同意を取得する（詳細は「11.2 被験者への説明と同意の取得」参照）。

6.2 スクリーニング

同意取得から登録時までに必要な検査及び調査を実施する（「6.6 観察・検査スケジュール」参照）。検査時期の許容範囲は、検査の侵襲等を考慮して、治験責任医師又は治験分担医師が被験者の病状に変化がないと判断できる場合に限り、血液検査、心臓MRI及び負荷心筋シンチグラフィーについては同意取得日から84日前まで、心臓カテーテル検査については同意取得日から3年前まで可とする。また、治験ノートを同意取得時に被験者に配布し、同意取得日から登録予定日までの間、被験者の硝酸薬使用回数／日ならびに胸痛回数／日を28日間以上（同意取得翌日から登録前日まで、全体で記載率70%以上）記載する。

6.3 適応評価委員会による選択基準(4)の適否判断

適応評価委員会は、治験責任医師又は治験分担医師から送付された被験者のデータについて、選択基準(4)の適否を判断し、治験責任医師又は治験分担医師にFAX等で適格性判定結果を報告する。手順については別途定める。

6.4 被験者登録

(1) 治験責任医師又は治験分担医師は、適応評価委員会による選択基準(4)の適否、被験者により28日間以上記載された治験ノート（記載率70%以上)、ならびに選択基準及び除外基準に基づき、被験者の適格性を判定し被験者登録する。

(2) 登録及び治験治療群の割付け

1) 治験責任医師又は治験分担医師は、被験者登録票に必要事項を記入し、適応評価委員会の適格性判定結果とともに登録センターにFAXにて、治験治療初回実施2日前までに送付する（治験責任医師又は治験分担医師の指導のもと治験協力者が行ってもよい)。

2) 登録センターは、被験者登録票により適格と判定された場合には、適格であることと登録番号が記載された適格性確認連絡票を発行し、FAX にて治験責任医師又は治験分担医師に送付、適格性確認連絡票及び被験者登録票の写しを治験調整医師に送付する。

3)適格性確認連絡票を受け取った治験責任医師又は治験分担医師は、予め治験責任医師に指名された治験治療担当医師に登録番号を伝える。治験治療担当医師は、事前に配布された割付け表から、その登録番号に割付けられている割付け群（実治療群、プラセボ群）を確認する。なお、治験治療担当医師は治験治療が適切に行われていることを確認する者を、可能な限り、医療従事者の中から指名する。指名された医療従事者は割付け群を知ることができる。割付け群については、治験治療担当医師及び指名された医療従事者のみが知ることとし、治験終了後、治験調整医師よりキーオープンの許可が出るまで割付け表ならびに各被験者の割付け群を他者へ漏らしてはならない。

4)選択基準に合致しない、あるいは除外基準に抵触し、登録に至らなかった場合は、登録センターより不適格であることとその理由を記載した適格性確認連絡票を治験責任医師又は治験分担医師へ送付する。不適格となった症例は、不適格症例として治験を終了する。

5) 治験責任医師又は治験分担医師は、被験者の登録が完了するまでは当該治験で規定された治療を開始してはならない。なお、事後登録や二重登録は許容されない。事後登録や二重登録が判明した場合には、治験責任医師又は治験分担医師は登録センターに速やかに連絡することとする。

(3) 中止時

1) 登録後、被験者が「4.3　中止基準」に該当したことが判明した場合や、その他の理由にて治験を中止する場合、治験責任医師又は治験分担医師は速やかに登録センターに症例中止連絡票をFAXにて送付し、治験を中止する（治験責任医師又は治験分担医師の指導のもと治験協力者でも可)。

2）登録センターは、症例中止連絡票を受領後、記載内容の確認を行った後に症例中止確認連絡書を治験責任医師又は治験分担医師に送付する。また、症例中止連絡票及び症例中止確認連絡書の写しを治験調整医師へ送付する。

(4) 再登録

治験治療実施前に治療中止となった被験者は、再登録を可能とする。この場合、(1)～(2)に示す手順を再度行うこととする。また、再登録の場合も、被験者識別番号は同一のものを使用することとする。再登録の場合は、治験責任医師により選択基準に合致し除外基準に抵触しない状態であると判断されれば、適応評価委員会の再評価は不要とする。

【登録先】

登録センター（東北大学病院臨床試験データセンター）

FAX：022-717-7580　　TEL：022-717-7137

登録時間：月～金曜日　9:00～17:00（祝祭日、年末年始は除く）

適応評価委員会	治験責任医師等	登録センター	治験調整医師
	被験者の選定<br>文書による同意取得<br>↓<br>スクリーニング検査<br>治験ノート記録<br>（28日以上）		
選択基準（4）の適否 ←→ CROを仲介	選択基準（4）の判定<br>↓<br>適格性（選択・除外基準）の判定<br>↓<br>登録票作成<br>↓		
	登録票及び適格性判定結果 → FAX	登録票及び適格性判定結果を受領<br>↓<br>適格性の確認	
	問い合わせ ←→ FAX	↓<br>適格<br>↓<br>登録の可否<br>↓<br>↓	
	適格性確認連絡票受領 ← FAX	適格性確認連絡票作成<br>*＜不適格の場合も確認連絡する＞*	→ 登録票及び適格性確認連絡票の写しを受領
	↓<br>↓<br>治験治療担当医師に登録番号を伝える<br>↓<br>割付け表から割付群を確認<br>（治験治療担当医師のみ）		

6.4.1 盲検性の保持の手順

当該治験は被験者・評価者盲検にて実施されることから、盲検性を保持するため下記の手順を遵守する。

(1)治験実施医療機関における治験治療担当医師の指名

治験実施医療機関において、治験責任医師は治験治療担当医師を指名する。治験治療担当医師は、治験分担医師から選出する。治験治療担当医師のみが、その医療機関内における被験者の割付け群（実治療群、プラセボ群）を知ることが出来る。また、治験治療担当医師は治験治療のみを実施し、治験治療以外の治験結果に影響を及ぼす業務（被験者の診察、症例報告書の記載、検査結果の判定等）を行うことは出来ない。なお、治験治療担当医師は治験治療が適切に行われていることを確認する者を、可能な限り、医療従事者の中から指名する。指名された医療従事者は割付け群を知ることができる。

(2)割付け

1)割付け方法は、CCS クラススコア（「Ⅱ」、「ⅢまたはⅣ」）、1 週間あたりのニトロ使用頻度（「4 回/週以下」、「5 回/週以上」）、左室駆出率（「40%以上」、「40%未満」）、施設を割付け因子とし実治療群とプラセボ治療群の 2 群へランダムに割付けする。

2)当該治験の統計解析責任者により割付け表が作成され、治験参加施設の治験治療担当医師へ配布される。割付け方法と割付け表の作成に関する詳細は、別途手順書で定める。

3)割付け表は治験治療担当医師及び治験治療担当医師が指名した医療従事者のみ閲覧することが可能であり、他者（治験責任医師又は治験分担医師、被験者、その他医療従事者等）へ開示してはならない。また、治験調整医師の許可があるまで、治験治療担当医師のみが開錠できる保管庫に割付け表を保管する。

4)治験治療担当医師は、登録センターから FAX にて送付された、適格性確認連絡票に記載されている登録番号に割付けられている群（実治療群、プラセボ群）を割付け表から確認する。

(3)治験治療

治験治療は治験治療担当医師のみが行い、当該治験期間に各治験実施医療機関で行われる治験治療を全て実施する。緊急時を除き、治験治療中に治験責任医師又は治験治療担当医師以外の治験分担医師、その他の医療従事者が立ち会うことは許されない。なお、治験治療担当医師が指名した医療従事者は立ち会うことができる。

6.4.2 キーオープン

(1) キーオープン

各被験者の割付け群（実治療群、プラセボ群）は、原則、治験が終了し、全参加治験実施医療機関の症例データが固定された後、治験調整医師の指示によりキーオープンを行う。

(2)緊急時のキーオープン

治験実施期間中であっても、緊急時などキーオープンが必要な場合は、治験責任医師の指示にて個々の被験者の割付け群（実治療群、プラセボ群）を知ることが出来る。その場合は、治験治療担当医師が該当する被験者の割付け群のみを開示する。緊急時のキーオープンの際は、治験責任医師は治験調整医師へ、可能な限り事前に状況を報告しキーオープンする理由を伝える。

6.5 治験機器による治療

6.5.1 治療手順

登録後、被験者・評価者盲検にて初回の当該治験機器による治療を実施する。登録は、治験治療初回実施-14～-2 日以内に行う。治験治療初回実施後 84 日の観察検査 3 の検査・観察を実施し、その後、28 日以内に実治療を行う。治験治療及び実治療のいずれも同じ手順にて超音波照射（プラセボ群は治験治療では超音波照射を行わない）を 1 日 3 回で 1 セットとし、隔日に合計 3 セットを実施する。

(1)治療前日までの準備

原則、治療前日には入院する。

(2)治療当日（治験治療又は実治療実施第 0 日、第 2 日、第 4 日）

1)血圧、体温測定は治療の前後で実施する。

2)画像上にて虚血領域と周囲臓器との位置関係を把握する。

(3)治験治療又は実治療の中止

治験治療又は実治療を中止する際は、「6.6 観察・検査スケジュール」に則って可能な限り中止時の観察・検査を行う。

(4) 治験治療実施前に治療中止となった場合、再登録を可能とする条件は下記の場合とする。

1)手技継続困難な器具の破損等による超音波照射不能の場合

2)手技継続困難な有害事象が発生した場合

3)その他、術者が治験治療前に手技を中止すべきと判断した場合

6.5.2 治験機器使用手順

治験治療及び実治療の場合、治験責任医師に指名された（割付けを知る）治験治療担当医師が治療を実施する。被験者を仰臥位とし、超音波プローブを前胸壁に当てて心臓を観察し、虚血部位を描写する。この時点で治療用の超音波発生条件に設定を変更することにより、描写範囲が治療範囲となる。虚血部位の同定は、複数の画像診断の結果をもとに行う。一断面につき 20 分±1 分照射を行い、5 分以上間隔を空け、心基部から心尖部の計 3 断面照射する。治療時の超音波の設定条件は、培養細胞と動物実験による基礎データをもとに決定した（0.25 W/cm^2, 1.875MHz, 矩形 32 サイクル)。

プラセボ群の治験治療の場合は、上記治験治療及び実治療の手順に準ずるが超音波照射は行なわない。

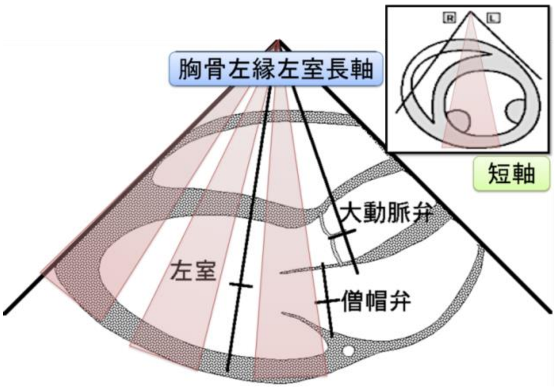

6.5.3 治験機器の使用上の注意

当該治験で用いる超音波の出力は、診断機器として使われる出力の上限程度であり、実験中も有害事象は認めず、安全性には問題ないと考えている。治療に用いる超音波の設定は、培養細胞を用いた実験から 32 サイクルが最適と考えられた。また、この設定条件で、大型動物（ブタ）においても、有効性・安全性が確認されている。治療に伴う超音波プローブの温度上昇は、国際的な規格値の範囲内であるが、治療前後にはプローブ接触部位の皮膚の状態を観察する。

6.6 観察・検査スケジュール

表 6-1 に従い、観察・検査を行う。

表 6-1　観察・検査スケジュール

		同意取得時	スクリーニング時[5]	登録時	治験治療											
					治験治療						観察期					
					治験治療直前	1回目		2回目		3回目		14日目	検査1	検査2	70日目	検査3[6]	検査4[9]
規定日		-86～-30日	-86～-2日	-14～-2日	0日		1日	2日	3日	4日	5日	14日	28日	56日	70日	84日	1年
許容範囲（日）					起点日		+3	+6	+9	+12	+14	±3	+7	±14	±3	±14	±28
同意取得		○															
登録				○													
超音波治療						○		○		○							
治験機器の調査[8]						○		○		○							
被験者背景			○														
体重・BMI[16]			○			○	○	○	○	○	○		△	△		○	○
血圧測定[16]			○			○[4]	○	○[4]	○	○[4]	○		△	△		○	○
体温測定						○[4]	○	○[4]	○	○[4]	○						
妊娠検査			○													○	
身体所見[16]			○			○	○	○	○	○	○		△	△		○	○
硝酸薬の使用記録[3]			←→			←	―	―	―	―	―	―	―	―	―	→	○
胸痛の発現記録[3]			←→			←	―	―	―	―	―	―	―	―	―	→	○
有害事象、主要心血管イベント[10]						←	―	―	―	―	―	―	―	―	―	→	○
CRCによる電話等での聴取[16]												○	△	△	○		
CCSクラススコア			○													○	
併用薬、併用療法調査			○	←	―	―	―	―	―	―	―	―	―	―	―	→	○
臨床検査[12]	血液検査[18]		○				○		○		○					○	
	血液生化学検査[18]		○				○		○		○					○	
	血液特殊検査[14]		○								○					○	
	安静時12誘導心電図		○				○		○		○					○	
	トレッドミル運動負荷心電図[17]	○														○	
	6分間歩行試験[15・17]	△														△	
画像検査[12]	胸部X線		○				○		○		○					○	
	心臓超音波検査		○													○	
	心臓MRI[13]	△														△	
	負荷心筋シンチグラフィー[1]	○														○	
	心臓カテーテル検査[2]	○															

		実治療													中止時[11]
		実治療							観察期						
		実治療前日[6・7]	1回目		2回目		3回目		14日目	検査1	検査2	70日目	検査3	検査5	
規定日		−1日	0日	1日	2日	3日	4日	5日	14日	28日	56日	70日	84日	1年	
許容範囲（日）[6・7]		−1	起点日	+3	+6	+9	+12	+14	±3	+7	±14	±3	±14	±28	
同意取得															
登録															
超音波治療			○		○		○								
治験機器の調査[8]			○		○		○								
被験者背景															
体重・BMI[16]		△	○	○	○	○	○	○		△	△		○	○	○
血圧測定[16]		△	○[4]	○	○[4]	○	○[4]	○		△	△		○	○	○
体温測定			○[4]	○	○[4]	○	○[4]	○							
妊娠検査															
身体所見[16]		△	○	○	○	○	○	○		△	△		○	○	○
硝酸薬の使用記録[3]		←												→	○
胸痛の発現記録[3]		←												→	○
有害事象、主要心血管イベント[10]		←												→	○
CRCによる電話等での聴取[16]									○	△	△	○			
CCS ｸﾗｽｽｺｱ													○		○
併用薬、併用療法調査		←												→	○
臨床検査[12]	血液検査			○		○		○					○		○
	血液生化学検査			○		○		○					○		○
	血液特殊検査[14]							○							
	安静時12誘導心電図			○		○		○					○		○
	ﾄﾚｯﾄﾞﾐﾙ運動負荷心電図												○		
	6分間歩行試験[15]												△		
画像検査[12]	胸部X線			○		○		○					○		○
	心臓超音波検査												○		○
	心臓MRI[13]												△		
	負荷心筋ｼﾝﾁｸﾞﾗﾌｨｰ[15]												○		
	心臓ｶﾃｰﾃﾙ検査														

○：必須とする検査項目。

△：可能な限り実施する検査項目。

1 症状に変化がない場合、同意取得日から 84 日前までの結果を使用可能とする。実治療観察期検査 3 は必須としない。

2 症状に変化がない場合、同意取得日から 3 年前までの結果を使用可能とする。また、合併症などのため施行困難な場合は、他の検査所見を参考に判断する。

3 同意取得日から治験ノート記載を開始し、記録記載が可能な患者だけを登録する。

4 治療の前後で測定する。

5 スクリーニング時検査のトレッドミル運動負荷心電図、6 分間歩行試験、心臓 MRI については、治験治療初回実施前までに実施すればよい。

6 観察期検査 3（治験治療初回実施後 84 日）終了後 28 日以内に実治療初回を実施する。

7 実治療前日は必須としない。実施しない場合は、実治療実施前に当該検査を実施し、実治療実施の可否を評価する。また、治験治療から実治療を継続し、観察期検査 3 と実治療前日が同日の場合、観察期検査 3 の検査・観察項目を実施する。

8 治験機器の調査は、6.6.2 調査・検査内容(21)を実施する。

9 治験治療検査 4 は、実治療に移行しなかった被験者に行う。

10 主要心血管イベント：MACE（心血管死、非致死性心筋梗塞、入院を要する不安定狭心症・心不全）

11 治験中止時は、可能な限り規定された検査・観察を実施する。

12 同意取得から実治療観察期検査 3 まで及び中止時に行う臨床検査及び心臓カテーテル検査以外の画像検査は、原則同一の医療機関で実施する。

13 撮像可能な場合、同意取得日から 84 日前までの結果を使用可能とする。心臓 MRI 撮像は必須としない。

14 血液特殊検査は、血漿を冷凍保管し、中央検査を実施する。なお、やむを得ない事情により、各治療 3 回目翌日の実施が困難な場合は、各治療 3 回目終了後同日にも実施可能とする。

15 6 分間歩行試験、実治療観察期検査 3 の負荷心筋シンチグラフィーは必須としない。

16 観察期検査 1、観察期検査 2 は必須としない。CRC による電話等での聴取は、観察期検査 1、観察期検査 2 に受診ができない被験者に対して行う。

17 症状に変化がない場合、同意取得日から 1 年前までの結果を使用可能とする。

18 スクリーニング時の血液検査及び血液生化学検査については、症状に変化がない場合、同意取得日から 84 日前までの結果を使用可能とする。

6.6.1 各観察･検査ポイントの規定日及び許容範囲

安全性・有効性の確認のため、「6.6　観察・検査スケジュール」を下記の規定日及び許容範囲内に実施する。許容範囲から外れた場合は、当該検査日のデータを欠測とする。

観察・検査ポイント		規定日	許容範囲
同意取得		－	－86～－30 日
スクリーニング時		－	－86～－2 日
登録時		－	－14～－2 日
治験治療	**治験治療**		※治験治療の 0～5 日目までを 14 日間以内で実施する
	1 回目（0 日目）	0 日目	起点日（0 日）
	1 日目	1 日目	＋3 日
	2 回目（2 日目）	2 日目	＋6 日
	3 日目	3 日目	＋9 日
	3 回目（4 日目）	4 日目	＋12 日
	5 日目	5 日目	＋14 日
	**観察期**		
	14 日目	14 日目	±3 日
	検査 1（28 日目）	28 日目	＋7 日
	検査 2（56 日目）	56 日目	±14 日
	70 日目	70 日目	±3 日
	検査 3（84 日目）	84 日目	±14 日
実治療	実治療前日	－1 日目	－1 日
	**実治療**		※実治療の 0～5 日目までを 14 日間以内に実施する
	1 回目（0 日目）	0 日目	起点日（0 日）
	※検査 3（84 日目）から 28 日以内に実施		
	1 日目	1 日目	＋3 日
	2 回目（2 日目）	2 日目	＋6 日
	3 日目	3 日目	＋9 日
	3 回目（4 日目）	4 日目	＋12 日
	5 日目	5 日目	＋14 日
	**観察期**		
	14 日目	14 日目	±3 日
	検査 1（28 日目）	28 日目	＋7 日
	検査 2（56 日目）	56 日目	±14 日
	70 日目	70 日目	±3 日
	検査 3（84 日目）	84 日目	±14 日
検査 4（1 年目）		治験治療 0 日目から 365 日目	±28 日
検査 5（1 年目）		実治療 0 日目から 365 日目	±28 日
中止時		－	速やかに規定された観察・検査を実施する

6.6.2 調査･検査内容

各調査･検査の観察日が治験治療と同日の場合は、治験治療後に規定がある場合を除き、治験治療前に実施する。また、治験治療期間から実治療への移行は、28 日以内とする。心臓カテーテル検査以外の検査は治験実施医療機関で検査したものを採用する。

なお、適応評価委員会での判断項目とするのは、心臓カテーテル検査（画像・所見)、病歴である。

(1)被験者背景

原疾患とは、被験者が罹患している疾患のうち、虚血性心疾患（狭心症、心筋梗塞等）とする。また、原疾患の他に、治験機器による治験治療初回実施時点で罹患している疾患を合併症とし、過去に罹患し治験機器による治験治療初回実施時点で治癒している疾患を既往歴とする。既往歴は治験治療初回実施前 1 年間を対象とするが、悪性腫瘍の既往歴及び手術歴（手術の有無、手術名、手術日）は治験治療初回実施前 5 年間を対象とする。これらは過去の診察データから調査する。

[調査項目]性別、生年月日、身長、原疾患、既往歴・合併症の有無及び疾患名、既往歴・合併症に心疾患を含む場合は治療歴の有無及び内容、左室駆出率（心臓超音波検査、心臓 MRI で評価)

[調査時期]同意取得～治験治療初回実施前まで

[CRF 記載内容]性別、生年月日、身長[cm](整数)、原疾患（狭心症、心筋梗塞、その他)、既往歴・合併症の有無及び疾患名、心疾患治療歴：PCI、CABG、PMI（CRT、ICD を除く)、CRT（CRT-P・CRT-D)、ICD、その他、左室駆出率[%]（心臓超音波検査で評価)

(2)体重、BMI

[検査項目]体重、BMI

[検査時期]スクリーニング時、各治療実施日と翌日、実治療初回実施前日、各治療初回実施後 28 日（可能な限り実施)、56 日（可能な限り実施)、84 日、実治療初回実施後 1 年、中止時、治験治療観察期 検査 4（治療実施後に中止し、可能な場合に実施)

[CRF 記載内容]体重［kg］（小数点第 1 位まで記載)、BMI（小数点第 1 位まで記載)

(3)血圧

[検査項目]収縮期血圧、拡張期血圧（いずれも入院では臥位、外来では坐位で測定)

[検査時期]スクリーニング時、各治療実施日と翌日、実治療初回実施前日、各治療初回実施後 28 日（可能な限り実施)、56 日（可能な限り実施)、84 日、実治療初回実施後 1 年、中止時、治験治療観察期 検査 4（治療実施後に中止し、可能な場合に実施)

[検査方法]被験者を臥位あるいは坐位で 5 分間安静にさせた後、測定する。3 回測定し、3 回の平均値を記録する。治療実施日は、治療の前後で実施する。

[CRF 記載内容]収縮期圧、拡張期圧[mmHg]（いずれも整数)

(4)体温

[検査項目]体温

[検査時期]各治療実施日と翌日

[検査方法]腋下にて測定する。治療実施日は、治療の前後で実施する。

[CRF 記載内容]体温［℃］（小数点第 1 位まで記載）

(5)妊娠検査

[検査項目]妊娠反応検査

[検査時期]スクリーニング時、治験治療初回実施後 84 日

[検査方法]妊娠可能*な女性を対象とし検査キットで行う。妊娠の可能性がない患者はその旨を症例報告書へ記載した上で検査を免除できる。

妊娠可能*：外科的に不妊（例：卵管結紮、卵巣又は子宮摘出等）の状態でない、あるいは閉経（1 年以上自然な無月経であることが確認されていること）をむかえていない。

[CRF 記載内容]妊娠の有無又は不要、妊娠検査を不要と判断した理由

(6)身体所見

[調査項目]心不全所見の有無

[調査時期]スクリーニング時、各治療実施日と翌日、実治療初回実施前日、各治療初回実施後 28 日（可能な限り実施）、56 日（可能な限り実施）、84 日、実治療初回実施後 1 年、中止時、治験治療観察期 検査 4（治療実施後に中止し、可能な場合に実施）

[調査方法]心不全所見は Framingham 基準に従い、大症状 2 つあるいは大症状 1 つ及び小症状 2 つ以上を心不全と診断する。

[CRF 記載内容]心不全所見の有無

表 6-2　Framingham 基準

大症状	・発作性夜間呼吸困難または起座呼吸<br>・頸静脈怒張<br>・肺ラ音<br>・心拡大<br>・急性肺水腫<br>・拡張早期性ギャロップ（Ⅲ音）<br>・静脈圧上昇（16cmH_2O 以上）<br>・循環時間延長（25 秒以上）<br>・肝頸静脈逆流
小症状	・下腿浮腫<br>・夜間咳嗽<br>・労作性呼吸困難<br>・肝腫大<br>・胸水貯留<br>・肺活量減少（最大量の 1/3 以下）<br>・頻脈（120/分以上）
大症状あるいは小症状	・　5 日間の治療に反応して 4.5kg 以上の体重減少があった場合，それが心不全治療による効果ならば大症状 1 つ，それ以外の治療ならば小症状 1 つとみなす

(7)硝酸薬使用記録（治験ノート）

［調査項目］硝酸薬使用回数

［調査時期］同意取得後登録まで（同意取得翌日から登録前日まで 28 日間以上とする)、治験治療初回実施後から実治療初回実施後 84 日まで、実治療初回実施後 1 年から前 28 日間以上、中止時、治験治療観察期 検査 4 から前 28 日間以上(治療実施後に中止し、可能な場合に実施)

なお、検査 4 及び検査 5 に該当する時期の治験ノートの記載忘れが発生しないよう、被験者への説明を適切な時期に行なうこと。

［調査方法］硝酸薬の内服（舌下）や吸入の頻度を被験者が治験ノートに記載する。硝酸薬使用は、1 錠あるいは 1 噴霧を 1 回とする。

ニトログリセリン、硝酸イソソルビド等硝酸薬の 1 週間当たりの平均使用量は、同意取得翌日から登録前日までの記録（28 日間以上）と、治験治療及び実治療初回実施後 84 日間、ならびに実治療初回実施後 1 年から前 28 日間のそれぞれの期間で算出する。記載内容は、登録時、治験治療又は実治療初回実施後 84 日には受診時に確認を行う。また、治験治療又は実治療初回実施後 14 日、70 日に担当 CRC が電話等で聞き取りを行

う。治験治療又は実治療初回実施後 28 日、56 日は、受診時の確認が望ましいが、困難な場合は医師あるいは担当 CRC が電話等で聞き取りを行う。

[治験ノート記載内容]胸痛時に使用している頓用の硝酸薬名、毎日の硝酸薬使用回数（正の字又は数字で回数を記載する。使用しなかった場合、「なし」又は「0」と記載する）

(8)胸痛記録（治験ノート）

[調査項目]胸痛回数

[調査時期]同意取得後登録まで（同意取得翌日から登録前日まで 28 日間以上とする）、治験治療初回実施後から実治療初回実施後 84 日まで、実治療初回実施後 1 年から前 28 日間以上、中止時、治験治療観察期 検査 4 から前 28 日間以上（治験治療実施後に中止し、可能な場合に実施）

[調査方法]治験ノートに被験者が毎日の胸痛回数を記録する。胸痛は強弱の程度等は問わず、自覚のあったものを 1 回として記録する。

[治験ノート記載内容]毎日の胸痛回数（正の字又は数字で回数を記録する。自覚しなかった場合は「なし」又は「0」と記載する。）

(9)CCS クラススコア

[検査項目]CCS クラススコア

[検査時期]スクリーニング時、各治療初回実施後 84 日、中止時

[検査方法]下記の表 6-3 に従って、狭心症の自覚症状について 4 段階で判定し記載する。同意取得後登録までの間、及び治験治療ならびに実治療初回実施後 56 日検査から同 84 日検査までの間の日常生活から、それぞれ判断する。

[CRF 記載内容]CCS クラススコア（Ⅰ、Ⅱ、Ⅲ、Ⅳ）

表 6-3　CCS クラススコア

Ⅰ	日常の身体活動では狭心症発作を起こさない。例）通常の歩行や階段上昇ができる。
Ⅱ	日常生活にわずかに制限がある。例）200m を超える平地歩行で狭心症発作を生じる。
Ⅲ	著しく日常生活が制限される。例）100m～200m の平地歩行で狭心症発作を生じる。
Ⅳ	あらゆる動作で症状が出現し、安静時にもみられる。

(10)併用薬・併用療法

併用薬・併用療法は、同意取得後、実治療初回実施後 1 年（中止又は治験治療観察期 検査 4） までに使用した全ての薬剤又は実施した全ての治療、処置を指す。

[調査項目]全ての使用薬剤、実施した全ての治療、処置名

[調査時期]同意取得時から実治療初回実施後 1 年目まで（中止時又は治験治療観察期 検査 4）

[CRF 記載内容]併用薬の薬剤名（商品名）、投与量・単位（／日、／週、頓用、その他）、使用目的、使用開始日、終了（中止）日又は継続中

併用療法名、併用療法の目的、併用療法開始日、終了（中止）日又は継続中

(11)血液検査

[検査項目・検査時期]

〈スクリーニング時、各治療初回実施後 84 日、中止時〉

①一般血液検査：白血球数、赤血球数、血色素量、ヘマトクリット値、血小板数

②血液生化学検査： 総ビリルビン、AST（GOT）、ALT（GPT）、LDH、BUN、クレアチニン、尿酸、Na、K、Cl、総蛋白、アルブミン、CK、中性脂肪、総コレステロール、HDL コレステロール、LDL コレステロール、血糖値、HbA1c、CRP、BNP

検査時期の許容範囲：スクリーニング時については、病状に変化がない場合に限り、同意取得日から 84 日前までに実施したデータを使用可とする。

〈各治療実施日翌日〉

①一般血液検査：白血球数、赤血球数、血色素量、ヘマトクリット値、血小板数

②血液生化学検査： AST（GOT）、ALT（GPT）、LDH、BUN、クレアチニン、Na、K、Cl、CK、CK-MB、CRP

[検査方法]空腹時検査を原則とする（採血量：7～14 mL）。

[CRF 記載内容]各検査値

(12)血液特殊検査

[検査項目]バイオマーカー

[検査時期]スクリーニング時、各治療 3 回目翌日、治験治療初回実施後 84 日

[検査方法]空腹時検査を原則とする（採血量：12 mL）。別途同意を取得する。

[CRF 記載内容]血液採取の有無

(13)安静時 12 誘導心電図

[検査項目]安静時 12 誘導心電図、心拍数

[検査時期]スクリーニング時、各治療実施日翌日、各治療初回実施後 84 日、中止時

[検査方法]被験者を仰臥位にして測定を行う。

[CRF 記載内容]調律の異常の有無（洞調律、心房細動、ペーシング、その他）、心拍数［回／分］、急性虚血性変化の有無

(14)トレッドミル運動負荷心電図

[検査項目]トレッドミル運動負荷心電図

[検査時期]同意取得～治験治療初回実施直前まで、各治療初回実施後 84 日

[検査方法]ベルト上で歩行を行う。2～3 分ごとにベルトの傾斜と速度を変化させ、徐々に負荷をかけていく。症状や兆候が出現した時点又は最大心拍数（220－年齢）の 80～90％に達した時点で中止する。ただし、上記は推奨する方法とし、観察期間を通して検

査方法を統一すること、負荷を漸増することを遵守すれば、必ずしも上記の方法に従わなくてもよい。
負荷前後及び運動中の 12 誘導心電図を測定し、0.1mV 以上の ST 低下又は ST 上昇を認めた時点の運動負荷量[W【Mets】]を記録する。心電図変化が認められなかった症例は、最大運動負荷量を記録する。

[CRF 記載内容]心電図変化出現時、症状出現時の運動負荷量[W【Mets】、時間【分】]、心電図変化が認められなかった時の最大運動負荷量[W【Mets】、時間【分】]、心電図変化出現の有無、症状出現の有無と内容（胸痛、呼吸困難、動悸、下肢疲労、その他）

(15)6 分間歩行試験（可能な限り実施）

[検査項目]6 分間歩行試験

[検査時期]同意取得～治験治療初回実施直前まで、各治療初回実施後 84 日

[検査方法]平坦な室内（各実施医療機関内の廊下など）を 6 分間できるだけ早く歩行し、胸痛の出現なしに歩行できた距離を記録する。途中で息切れ又は疲労した場合は、ペースを落としたり休むことは可能である。

[CRF 記載内容]歩行距離［m］(整数)、症状出現の有無と内容（胸痛、呼吸困難、動悸、その他）

(16)胸部Ｘ線

[検査項目]胸部Ｘ線

[検査時期]スクリーニング時、各治療実施日翌日、各治療初回実施後 84 日、中止時

[検査方法]撮像条件は被験者が立位、胸部正面像とする。心房や心室の拡大、体/肺血管の走行や形態異常などの情報から心拡大、動脈硬化の進行具合、狭心症及び心筋梗塞の可能性を判断する。心拡大については心胸郭比(%)を用いて評価を行う。

[CRF 記載内容]心胸郭比[%]、肺うっ血の有無、胸水の有無、治験治療実施後及び実治療実施後のみ気胸の有無

(17)心臓超音波検査

[検査項目]心臓超音波検査

[検査時期]スクリーニング時、各治療初回実施後 84 日、中止時

[検査方法]心筋壁運動及び心臓の形態の異常を検出する。スクリーニング時において、心筋壁の厚さ及び動きから虚血領域の特定を行い、虚血部位を記録する。また、心内血栓の有無についても評価を行う。各治療初回実施後 84 日検査では、虚血領域についてスクリーニング時と比較して評価を行う（領域範囲の変化や心臓の動きなど)。

[CRF 記載内容]壁運動異常（無・前壁・前側壁・側壁・後側壁・後壁・下壁・心室中隔・心尖部)、左室拡張末期径[mm]、左室収縮末期径[mm]、左室駆出率[%]、拡張末期中隔壁

厚[mm]、拡張末期後壁厚[mm]、心嚢液貯留の有無、虚血領域の変化や心臓の動きの変化の有無（各治療初回実施後 84 日目検査時）

(18)心臓 MRI（可能な限り実施）

有効性の評価に関しては中央判定を実施する。中央判定に関する詳細は中央判定に関する手順書に定める。

[検査項目]心臓 MRI

[検査時期]同意取得～治験治療初回実施直前まで、各治療初回実施後 84 日

検査時期の許容範囲：検査の侵襲等を考慮して、病状に変化がない場合に限り、同意取得日から 84 日前までに実施したデータを使用可とする。

[検査方法]撮像条件については別途手順を定める。左室駆出率[%]、左室拡張末期容積[mL]及び左室一回拍出量[mL]等について記録する。なお、心臓 MRI を施行できない症例においては、実施しなくともよい。

[CRF 記載内容]検査実施日

[中央判定内容] 左室拡張末期容積[mL]、左室収縮末期容積[mL]、左室駆出率[%]、左室一回拍出量[mL]

(19)負荷心筋シンチグラフィー

有効性の評価に関しては中央判定を実施する。中央判定に関する詳細は中央判定に関する手順書に定める。

[検査項目]負荷心筋シンチグラフィー

[検査時期]同意取得～スクリーニング時、治験治療初回実施後 84 日、実治療初回実施後 84 日

検査時期の許容範囲：検査の侵襲等を考慮して、病状に変化がない場合に限り、同意取得日から 84 日前までに実施したデータを使用可とする。

[検査方法]撮像条件については別途手順を定める。アデノシンを用いた薬物負荷心筋シンチグラフィーを行い、安静時及び薬物負荷時の心筋シンチグラフィーを比較する。

[CRF 記載内容] 検査実施日

[中央判定内容] 虚血領域（前壁・前側壁・側壁・後側壁・後壁・下壁・心室中隔・心尖部）、SSS(Summed Stress Score)、SRS(Summed Rest Score)、SDS(Summed Difference Score)

(20)心臓カテーテル検査

適応評価委員会での判断項目とする。

[検査項目]心臓カテーテル検査

[検査時期]同意取得～スクリーニング時

検査時期の許容範囲：検査の侵襲等を考慮して、病状に変化がない場合に限り、同意取得日から 3 年前までに実施した検査結果を使用可とする。（なお、治験実施医療機関以

外の他の医療機関で実施した検査結果を使用してもよい。その場合は、画像等の検査結果が提供されており、治験実施医療機関にて適切に保管されていることとする。)

[検査方法]造影剤による冠動脈の形態学的異常（狭窄、閉塞など）を検出する。狭窄率（%)、狭窄部位及び狭窄の状態について記録する。

[記載内容]AHA 冠動脈区域分類による狭窄度[%]、側副血行路の有無、左室造影所見、コメント（治験実施医療機関の治験責任医師又は分担医師のコメントを記載する）

(21)治験機器の調査

[調査項目]

超音波診断の使用前点検(機器取扱い説明書)

装置本体表面の損傷、電源プラグ及び電源ケーブルの損傷、TV 画面に表示される日付及び時刻の確認

治療に伴う調査

使用するプローブ、プローブ表面及びプローブケーブル、プローブコネクタの損傷

使用するプローブでの動作確認、使用する治療モード(ソフト)

[調査時期]治験治療及び実治療実施前

[CRF 記載内容]動作前確認実施（実施/未実施)、操作上の不具合（あり/なし)、治験機器、付属品及び周辺機器の破損・不具合（あり/なし）

(22)有害事象（主要心血管イベント：MACE を含む）

[調査項目]治験治療初回実施後から実治療初回実施後 1 年までの有害事象及び主要心血管イベント：MACE について調査を行なう。

[調査時期]治験治療初回実施から治験治療及び実治療初回実施後 1 年目又は中止時

[CRF 記載内容]「7．安全性評価」を参照

(23)治験機器治療

[調査項目]治験機器治療開始時刻及び終了時刻

[調査時期]各治験治療及び各実治療

[CRF 記載内容]治験治療及び実治療実施の有無、治験機器治療日、治療開始時刻、治療終了時刻、コメント（機器脱落時間等）

6.6.3 中止後の検査・観察

登録後、治験を中止した場合はすべて中止症例として取り扱う。治験治療又は実治療を 1 回でも行った後に中止となった場合は、可能な限り、「中止時」の検査・観察項目を実施する。また、有害事象で追跡が必要な場合は、原則として回復又は軽快するまで経過を追うこととする。なお、被験者の同意を得た上で、可能な限り治験治療又は実治療初回実施後 1 年目(検査 4 又は検査 5)を実施する。

7. 安全性評価

7.1 臨床検査値

治験責任医師又は治験分担医師は、当該治験機器による治験実施後に測定した検査値及び測定値を、それぞれ同意取得後に検査した結果と比較し、異常変動の有無の判定を行う。臨床検査値の異常変動を判定する際は「National Cancer Institute Common Terminology Criteria for Adverse Events (NCI CTCAE v4.0)」を参考とし、該当する項目がない場合は、表7－1を参考にGradeを評価する。

臨床検査値異常が認められた場合、治験責任医師又は治験分担医師は以下の項目を参考として当該異常を有害事象とするか判断する。

(1) 臨床検査値異常によって治験機器による治療が延期又は中止された場合
(2) 臨床検査値異常を改善するため、薬物による介入や外科的介入が行われた場合
(3) 臨床症状との関連性が認められた場合
(4) 重篤な有害事象との関連が認められた場合
(5) 上記(1)～(4)以外で、治験責任医師又は治験分担医師が有害事象とすべきと判断した場合

表7-1 有害事象のGrade分類(重症度)基準

Grade	重症度
1	軽度。症状がない、又は軽度の症状がある。臨床所見又は検査所見のみ。治療を要さない。
2	中等度。最小限/局所的/非侵襲的治療を要する。年齢相応の身の回り以外の日常生活動作の制限*。
3	重症又は医学的に重要であるが、ただちに生命を脅かすものではない。入院又は入院期間の延長を要する。活動不能/動作不能。身の回りの日常生活動作の制限**。
4	生命を脅かす。緊急の処置を要する。
5	有害事象(AE)による死亡。

*身の回り以外の日常生活動作（instrumental ADL）とは食事の準備、日用品や衣類の買い物、電話の使用、金銭の管理などをさす。

**身の回りの日常生活動作（self care ADL）とは、入浴、着衣・脱衣、食事の摂取、トイレの使用、薬の服薬が可能で、寝たきりではない状態をさす。

7.2 有害事象

7.2.1 有害事象の定義

有害事象（主要心血管イベント：MACE含む）とは、当該治験機器による治療（プラセボ治療を含む）を実施した被験者及び術者等に生じたすべての好ましくない、又は意図しない疾病又は障害ならびにその徴候（臨床検査値の異常を含む）をいい、当該治験機器と

の因果関係の有無は問わない。有害事象の収集は、当該治験機器による治療以降、個々の被験者の観察期間終了又は中止時までの期間（追跡調査期間は除く）とし、重症度は「National Cancer Institute Common Terminology Criteria for Adverse Events（NCI CTCAE v4.0）」に従って判定する。また、該当する項目がない場合は、表 7-1 を参考に Grade を判定する。

7.2.2 有害事象の記載

有害事象名は、原則として診断名・疾患名（病名）で症例報告書に記載する。診断名・疾患名が特定できない場合や治験責任医師又は治験分担医師より診断名・疾患名としないことが妥当と判断された場合には、臨床症状又は徴候（臨床検査値異常を含む）を有害事象名として症例報告書に記載する。

治験責任医師又は治験分担医師は、発現したすべての有害事象に関し、有害事象名、程度（重篤、非重篤）及び重篤と判断した理由（7.2.3 重篤な有害事象（1）～（7）参照）、発現日、転帰日、処置、転帰（回復、軽快、回復したが後遺症あり、未回復、死亡）、当該治験機器との因果関係、コメント（因果関係と判定理由等）を症例報告書の有害事象欄に詳細に記載する。

7.2.3 重篤な有害事象

有害事象が下記のいずれかに該当する場合、重篤な有害事象として取り扱う。

（1）死亡した場合
（2）死亡につながる恐れのある場合
（3）永続的又は顕著な障害・機能不全に陥った場合
（4）障害につながる恐れのある場合
（5）治療のため入院又は入院期間の延長が必要とされる場合
（6）先天異常をきたすもの
（7）その他医学的に重篤な状態

なお、治験実施計画書で規定されている入院、治験参加前（同意取得前）より予定していた療法又は検査を治験実施中に実施することのみを目的とした入院（予定手術や検査等）、また、有害事象に伴う治療・検査の目的以外の入院（健康診断等）は重篤な有害事象として取り扱わない。

7.2.4 有害事象の重篤性の判定

有害事象の重篤性は、以下に分類される。

（1）重篤である
（2）重篤でない

7.2.5 有害事象の治療のために取られた処置

有害事象を治療するために被験者に対して取られた処置は以下に分類される。

(1) あり（記載内容：薬物療法、その他（詳細））※ありの場合は、併用薬・併用療法記載箇所に詳細情報を記載する
(2) なし

7.2.6 治験機器に対して取られた処置

有害事象が発現した場合に、当該治験機器に対して取られた処置は以下に分類される。

(1) なし
治験機器の使用を中止しなかった場合
(2) 使用休止
治験機器の使用を一旦中止し、その後使用を再開した場合
(3) 使用中止
治験機器の使用を中止した場合（治験期間中に再開することはなかった）

7.2.7 有害事象の転帰

有害事象の転帰は以下に分類される。

(1) 回復
有害事象が消失、又は元の状態まで戻っている
(2) 軽快
有害事象は完全に回復していないものの、ほぼ消失、又はほぼ元の状態に戻っている
(3) 回復したが後遺症あり
有害事象は元の状態まで回復したものの、後遺症が残っている
(4) 未回復
有害事象は継続中である
(5) 死亡
有害事象の結果、死亡した

個々の被験者の治験観察期間終了日の時点で未回復の有害事象については、可能な限り回復又は軽快となるまで追跡調査を行うこととするが、不可逆的な事象であった場合等、治験責任医師又は治験分担医師が追跡不要と判断した場合は、被験者の治験期間終了日をもって追跡終了とすることができる。その場合は、症例報告書のコメント欄に追跡不要と判断した理由を記載する。

また、有害事象は下記の表 7-2 の判定基準に従って当該治験機器との因果関係を 3 段階で判定し、症例報告書に記録する。表 7-2 に基づき、当該治験機器との関連性で「関連性は否定できない」「関連あり」のいずれかに該当したものを当該治験機器との因果関係が否定できない事象とみなす。なお、「関連なし」に該当する場合は、判断した理由を症例報告書のコメント欄に記載する。

表 7-2　治験機器との因果関係判定基準

関連なし	治験機器との関連が明らかに否定できる場合
関連性は否定できない	治験機器との関連が疑われる場合
関連あり	治験機器との関連が明らかに認められる場合

7.3 不具合

不具合とは、当該治験機器の仕様上の問題、当該治験機器の不良・故障、又は手技の問題等をいう。不具合情報は治験実施期間中に発生した情報を随時収集する。

7.3.1 不具合情報の記録

治験責任医師又は治験分担医師は当該治験機器に不具合が発生した場合、発生した不具合情報の内容を症例報告書へ記載する。不具合の安全度は表 7-3 に基づき判定する。

表 7-3　不具合の安全度

安全度 1	被験者及び術者等に影響を与える
安全度 2	被験者及び術者等に影響を与える可能性がある
安全度 3	被験者及び術者等に影響を与えない

7.4 有害事象及び不具合発生時の対応

(1) 治験責任医師又は治験分担医師は、有害事象又は不具合に対する治療が必要になったことを知った場合には、被験者にその旨を伝える。

(2) 治験責任医師又は治験分担医師は、有害事象又は不具合の発現に際しては適切な処置を施し、被験者の安全確保に留意し、その原因究明に努める。

(3) 治験責任医師又は治験分担医師は、発現した症状あるいは臨床検査値の異常変動について、原則として当該事象が消失又は治験開始前の状態に回復するまで、又は臨床上問題とならないと判断されるまで、可能な限り経過観察を継続し、その転帰を確認する。

7.5 重篤な有害事象及び重篤な有害事象につながる恐れがある不具合が発生した場合の報告

治験中の被験者において有害事象が発生し、治験責任医師又は治験分担医師が当該事象を重篤と判断した場合、以下手順に従い当該有害事象を取り扱う。

(1) 治験責任医師から治験実施医療機関の長、治験調整医師及び治験機器提供者への報告

治験責任医師は、因果関係に関わらず当該有害事象情報を可能な限り速やかに実施医療機関の長に報告するとともに、治験調整医師及び治験機器提供者に報告する。

(2) 治験調整医師による各実施医療機関の治験責任医師への通知

治験調整医師は、治験責任医師から入手した有害事象報告の内容を確認し、他の各実施医療機関の治験責任医師に当該有害事象情報を通知する。

(3) 治験責任医師と治験調整医師との協議

各実施医療機関の治験責任医師は、治験調整医師から入手した報告内容を確認し、必要に応じ治験責任医師と協議し、治験責任医師としての意見（厚生労働大臣への報告の必要性を含む）を治験調整医師に報告する。

(4) 効果安全性評価委員会の開催

治験調整医師が、当該重篤な有害事象が薬事法施行規則第 275 条で準用する第 273 条に規定される報告対象であると判断した場合は、効果安全性評価委員会を開催し、治験の継続の可否について判断を求める。効果安全性評価委員会が治験の継続の可否について判断した場合は、治験調整医師に通知し、治験調整医師により治験責任医師へ通知する。

(5) 厚生労働大臣への報告

治験責任医師が厚生労働大臣への報告が必要と判断した場合には、治験調整医師は指定の様式で報告書を作成し、独立行政法人医薬品医療機器総合機構（以下、総合機構という）に報告する。

(6) 実施医療機関の長への報告

治験責任医師は、他の実施医療機関において発生した重篤な有害事象について厚生労働大臣への報告がなされた場合には、治験調整医師より入手した（5）の報告書の内容を、可能な限り速やかに実施医療機関の長に報告する。

(7) 追加情報の入手時の対応

当該有害事象が発生した実施医療機関の治験責任医師は、当該事象に関する追加情報が得られた場合には、可能な限り速やかに実施医療機関の長に追加報告を行うとともに、治験調整医師及び治験機器提供者に報告する。当該追加情報の取り扱いは、上記（1）～（6）の手順に準ずることとし必要に応じ総合機構への報告等を行う。

7.6 新たな情報の提供

治験責任医師は、被験者及び術者等の安全性に悪影響を及ぼし、当該治験の実施に影響を与える、又は治験継続に関する治験審査委員会等の承認を変更する可能性のある情報を得た場合には、速やかに実施医療機関の長に文書で報告し、説明文書を改訂する必要があると認めた時は速やかに改訂する。

8. 有効性評価

8.1 主要評価項目

(1) 硝酸薬の週平均使用頻度

ニトログリセリン、硝酸イソソルビド等硝酸薬の 1 週間当たりの平均使用量は、28 日間の記録から評価する。同意取得翌日から登録前日までの 28 日間以上、治験治療及び実治療初回実施後 84 日間、ならびに実治療初回実施後 1 年の受診前 28 日間において、硝酸薬の内服(舌下)や吸入の頻度を被験者が治験ノートに記載する。記載内容は、登録時、治験治療又は実治療初回実施後 84 日には受診時に確認を行う。また、治験治療又は実治療初回実施後 14 日、70 日に担当 CRC が電話等で聞き取りを行う。治験治療又は実治療初回実

施後 28 日、56 日は、受診時の確認が望ましいが、困難な場合は医師あるいは担当 CRC が電話等で聞き取りを行う。

8.2 副次的評価項目

(1) 自覚症状（CCS クラススコア、胸痛の週平均発現頻度）

CCS クラススコアは、同意取得後登録までの間、及び治験治療ならびに実治療初回実施後 56 日検査から同 84 日検査までの間の日常生活から、それぞれ判断する。

胸痛の週平均発現頻度は、28 日間の記録から評価する。同意取得後登録までの 28 日間以上、治験治療及び実治療初回実施後 84 日間、ならびに実治療初回実施後 1 年から前 28 日間において、毎日の胸痛の回数を被験者が治験ノートに記載する。記載内容は、登録時、治験治療又は実治療初回実施後 84 日には受診時に確認を行う。また、治験治療又は実治療初回実施後 14 日、70 日に担当 CRC が電話等で聞き取りを行う。治験治療又は実治療初回実施後 28 日、56 日は、受診時の確認が望ましいが、困難な場合は医師あるいは担当 CRC が電話等で聞き取りを行う。

(2) 運動耐用能（トレッドミル運動負荷心電図、6 分間歩行試験）

トレッドミル運動負荷心電図では、12 誘導心電図で 0.1mV 以上の ST 低下又は ST 上昇を認めた時の運動負荷量を評価する。心電図変化が認められなかった症例では、最大運動負荷量を用いる。

6 分間歩行試験では、平坦な室内（各実施医療機関内の廊下など）を胸痛の出現なしに 6 分間で歩ける距離を評価する。

(3) 心筋血流（アデノシンを用いた薬物負荷心筋シンチグラフィー）

負荷心筋シンチグラフィーでは、治療領域における心筋虚血所見を評価する。

(4) 左室駆出率 40%以下の低心機能例における心機能（心臓超音波検査、心臓 MRI で評価)。

低心機能例（左室駆出率 40%以下）における心機能の変化を心臓超音波検査、心臓 MRI で評価する。なお、心臓 MRI の評価は、検査施行例のみ対象とする。

9. 統計解析

解析の主要な方針を以下の項目に示すが、より技術的で詳細な解析事項及び解析方法については、統計解析計画書に記載する。データ固定前に統計解析計画書を作成し、解析はデータ固定後に実施する。

個別の症例及びデータの取扱いについては、データ固定前の症例検討委員会において決定する。

9.1 解析対象集団

治験治療、実治療いずれの場合も、治療を 1 セット 3 回受けていない被験者については、解析対象から除外する。ただし、安全性評価については、治療を 1 回でも受けていれば解析対象とする。

9.2 有効性の解析

(1) 主要評価項目の解析

治療前後（同意取得後登録までの 28 日間、及び治験治療初回実施後 84 日目の検査前 28 日間）における硝酸薬の週平均使用頻度を評価する。平均値の変化、中央値の変化、及び改善した被験者の割合が、プラセボ治療群に比して実治療群において有意に高い場合、有効と判断する。

(2) 副次評価項目の解析

自覚症状、運動耐用能、心筋血流、低心機能例における心機能の各項目について、平均値の変化、中央値の変化、及び改善した被験者の割合が、プラセボ治療群に比して実治療群において有意に高い場合、有効と判断する。

9.3 安全性の解析

安全性解析対象集団を対象とした解析を実施する。

有害事象・重篤な有害事象の頻度集計を行う。また、有害事象・重篤な有害事象による治験治療の中止を離脱率として評価する。なお、治療の安全性については実治療初回実施後1年までの主要心血管イベント：MACE（心血管死、非致死性心筋梗塞、入院を要する不安定狭心症・心不全）の発生頻度を、今回と同様の症例を対象とした過去の論文報告との間接比較により評価する。その発生頻度が、40%を越えないことを確認する。

不具合の評価については、治験期間中（定期点検も含む）、被験者への使用の有無に関わらず当該治験機器において不具合の発生例数を調べる。

9.4 症例数の設定

目標登録症例数として以下の設定根拠に基づき80例（各群40例）と設定した。

<設定根拠>

症例数の設定根拠は、以下の通り。下川らが以前行った、虚血性心疾患患者を対象とした臨床試験において、低出力体外衝撃波治療前と治療 3 か月後における 1 週間あたりのニトログリセリン使用量の変化はそれぞれ平均 4.1 – 0.9 = 3.3 （回/週）であり、両群での差は 2(回/週)であった。また、両群で共通と仮定した 1 週間あたりのニトログリセリン使用量の変化の標準偏差は 2.4(回/週)であった。これらを参考に、主要評価項目の解析における検定の有意水準を 5%（両側）、検出力を 80%とした場合に、1 群あたり 24 例必要となる。脱落率を 10%程度考慮し、各群 30 例以上であれば十分と判断した。一方、安全性の評価として、主要心血管イベントの発生頻度を実治療後 1 年間にわたって検討することも考慮して、80 例（各群 40 例）と設定した。

東北大学病院では、年間約 250 例の経皮的冠動脈インターベンション、年間約 30 例の冠動脈バイパス術を行っている。しかし、バイパス血管の閉塞や新たな狭窄病変により心筋虚血領域が出現し、かつ経皮的冠動脈インターベンションや冠動脈バイパス術による治療が困難な例が、年間 5 例程度（1.8%）存在する。このような症例に対して超音波治療を加えるこ

とにより、症状の改善が期待される。対象となりうる症例のうち、他院からの紹介症例を含めて、1 施設あたり年間 10 例程度と見込んでいる。多施設で実施するため、フォローアップ期間も含めて、治験実施期間を 3 年間とした。

9.5 その他統計解析に関する事項

(1) 欠測値及び外れ値の取り扱い

1)欠測値

欠測値の取り扱いについては、統計解析計画書に記載する。最終的には、症例検討委員会で決定する。

2)外れ値

計画時においては外れ値の出現を想定しないが、必要に応じて、外れ値が解析に及ぼす影響を検討する。

(2) データ変換

データの分布に偏りが認められた場合は、必要に応じて適切なデータ変換を行う。

(3) 区間推定の信頼係数

区間推定の信頼係数は両側95%とする。

(4) 各評価、検査及び観察時期の許容範囲

「6.6.1 各観察・検査ポイントの規定日及び許容範囲」に記載した。

(5) その他

検定では有意水準を両側 5%とし、信頼区間は両側 95%信頼区間を用いる。

必要に応じて、探索的に追加解析を行う。

症例登録の進みが遅い場合、各群 30 例の時点で最終解析の実施を検討する。その場合、効果安全性評価委員会で実施の可否を審議する。

10. 治験の品質管理及び品質保証

治験の品質を保証するために、治験責任医師及び治験実施医療機関は、標準業務手順書に従い治験の品質管理を行う。

10.1 原資料等の直接閲覧

治験実施医療機関の長及び治験責任医師は、モニタリング、監査ならびに治験審査委員会及び国内外の規制当局による調査を受け入れ、原資料などのすべての治験関連記録を直接閲覧（複写含む）に供しなければならない。詳細は標準業務手順書に従う。なお、被験者は同意書に記名・押印又は署名することにより、直接閲覧を認めたことになる。

10.2 モニタリング

治験責任医師が指名するモニタリング担当者は、「モニタリングに関する手順書」に従い、治験実施計画書に記載されている全ての内容ならびに厚生労働省令第 36 号「医療機器

の臨床試験の実施の基準に関する省令（医療機器GCP)」に従って治験が実施されているかを確認するため治験実施医療機関へのモニタリングを定期的に行う。

10.3 監査

治験責任医師が指名する監査担当者は、「監査に関する手順書」に従い、治験実施医療機関及び開発業務受託機関において監査を実施する。

10.4 モニタリング及び監査への協力

治験責任医師及び治験実施医療機関の長ならびに開発業務受託機関の責任者は、モニタリング及び監査に協力をしなければならない。

10.5 治験責任医師保管書類

当該治験に関連して治験責任医師が保管すべき書類は、治験責任医師書類ファイルなどに保存し、治験責任医師がこのファイルを管理する。

11. 倫理及びGCP遵守

11.1 治験の倫理的実施

当該治験は、ヘルシンキ宣言の精神に基づいて実施する。また、薬事法第14条第3項及び第80条の2に規定する基準、「医療機器の臨床試験の実施の基準に関する省令（医療機器GCP)」等の関連法令通知及び治験実施計画書を遵守して実施する。

11.2 被験者への説明と同意の取得

(1) 治験責任医師は、被験者から治験への参加の同意を得るために用いる説明文書を作成し、必要な場合にはこれを改訂する。
(2) 作成又は改訂された当該文書は、あらかじめ治験審査委員会の承認を得る。
(3) 説明文書には、以下の事項を含むものとする。
  1) 治験が研究を伴うこと。
  2) 治験の目的。
  3) 治験責任医師又は治験分担医師の氏名、職名及び連絡先。
  4) 治験の方法。
  5) 予期される臨床上の利益及び危険性又は不便。
  6) 患者を被験者にする場合には、当該患者に対する他の治療方法の有無及びその治療方法に関して予測される重要な利益及び危険性。
  7) 被験者の治験への参加予定期間。
  8) 治験への参加は被験者の自由意思によるものであり、被験者又はその代諾者は、被験者の治験への参加を随時拒否又は撤回することができること。また拒否・撤回によって被験者が不利な扱いを受けたり、治験に参加しない場合に受けるべき利益を失うことはないこと。

9) 被検者の権利を放棄させるかその懸念のある記載又は治験責任(分担)医師、実施医療機関、治験依頼者の法的責任を回避するかその懸念のある記載をしてはならない。

10) モニター、監査担当者、治験審査委員会等及び規制当局が医療に係わる原資料を閲覧できること。その際、被験者の秘密は保全されること。同意文書に被験者又はその代諾者が記名・押印又は署名することによって閲覧を認めたことになること。

11) 治験の結果が公表される場合であっても、被験者の秘密は保全されること。

12) 被験者が治験及び被験者の権利に関してさらに情報の入手を希望する場合又は治験に関連する健康被害が生じた場合に照会すべき又は連絡をとるべき実施医療機関の相談窓口。

13) 治験に関連する健康被害が発生した場合に被験者が受けることのできる補償及び治療。

14) 治験に参加する予定の被験者数。

15) 治験への参加の継続について被験者又はその代諾者の意思に影響を与える可能性のある情報が得られた場合には、速やかに被験者又はその代諾者に伝えられること。

16) 治験への参加を中止させる場合の条件又は理由。

17) 被験者が費用負担をする必要がある場合にはその内容。

18) 被験者に金銭等が支払われる場合にはその内容（支払額算定の取り決め等)。

19) 被験者が守るべき事項。

20) 当該治験の適否等について調査審議を行う治験審査委員会の種類、各治験審査委員会において調査審議を行う事項その他当該治験に係る治験審査委員会に関する事項。

21) 治験審査委員会の手順書、委員名簿、委員会の議事要旨等の情報が公開され閲覧できる旨の記載。

22) その他、被験者の人権保護に関し必要な事項。

11.3 同意取得の方法

(1) 治験責任医師又は治験分担医師は被験者に対し、治験の参加に先立ち、治験審査委員会の承認を得た説明文書を使用して十分説明した後、自由意思による治験参加の同意を本人から文書で得る。

(2) 説明にあたっては、治験責任医師又は治験分担医師は被験者から同意を得る前に、被験者が質問する機会と、治験に参加するか否かを判断するのに必要な時間を与えなければならない。さらに、その際当該治験責任医師、治験分担医師又は補足説明者としての治験協力者は、すべての質問に対して被験者が満足するように答えなければならない。

(3) 同意説明文書には、説明を行った治験責任医師又は治験分担医師及び被験者が記名・押印又は署名し、各自日付を記入する。なお、治験協力者が補足的に説明を行った場合には、当該治験協力者も記名・押印又は署名し、日付を記入する。

(4) 治験責任医師又は治験分担医師は、記名・押印又は署名と日付が記入された同意文書の写し及びその他の説明文書を被験者が治験に参加する前に被験者に渡す。

(5) 同意文書の原本は実施医療機関で保存する。

11.4 同意説明文書の改訂

(1) 被験者の同意に関連し得る新たな重要な情報（通常、同意説明文書の改訂を必要とする情報）が得られた場合には、治験責任医師は速やかに当該情報に基づき同意説明文書を改訂し、治験審査委員会の承認を得る。

(2) 治験責任医師又は治験分担医師は、改訂された同意説明文書を用いて改めて説明し、治験への参加の継続について被験者から自由意思による同意を文書により得る。

治験責任医師又は治験分担医師は、新たに記名・押印又は署名と日付を記入した同意文書の写し及び説明文書を被験者に渡す。同意文書の原本を実施医療機関で保存する。

11.5 治験審査委員会(IRB)

当該治験は、治験の実施に先立ち、治験を行うことの適否について倫理的、科学的及び医学的妥当性の観点から、治験審査委員会の承認を得る。

11.6 被験者の人権保護に関する事項

治験責任医師又は治験分担医師は、被験者の選定にあたって、人権保護の観点から選択基準及び除外基準に基づき、被験者の健康状態、症状、年齢、同意能力、治験責任医師又は治験分担医師との依存関係、他の治験への参加の有無等を考慮のうえ、治験に参加を求めることの適否について慎重に検討する。

被験者の登録及び症例報告書における被験者の特定は被験者識別番号で行うとともに、治験の実施に係る原資料類及び被験者の同意説明文書等の直接閲覧、ならびに治験成績の公表においては、被験者の氏名、疾患等のプライバシー保護に十分配慮する。

11.7 データの二次利用について

当該治験で得られたデータについては個人識別情報とリンクしない形でデータを二次利用することがある。また、血液特殊検査で採取した血液（12 mL）については病態解明等の研究のため使用する目的で別途定めた手順書に従い各施設にて処理されたのち、中央測定機関に送付、保存、廃棄する。検体採取に関しては被験者の同意のもとにて行い、検体は匿名化されることとする。

12. 症例報告書

12.1 症例報告書の様式･構成

症例報告書（以下、CRF）は、下記の通り分割されたvisit型である。また、治験ノートについては、被験者が記載したものが発生するタイミングを下記に挙げる。

Visitタイトル	該当する観察・検査スケジュールのポイント	治験ノート
①スクリーニング時、登録時	同意取得時、スクリーニング時、登録時	スクリーニング時
②治験治療	1回目（0日目）、1日目、2回目（2日目）、3日目、3回目（4日目）、5日目	
③治験治療：14日目、検査1（28日目）	14日目、検査1（28日目）	治験治療1回目（0日目）～検査1(28日目）
④治験治療：検査2（56日目）	検査2（56日目）	検査1（28日目）～検査2（56日目）
⑤治験治療：70日目、検査3（84日目）	70日目、検査3（84日目）	検査2（56日目）～検査3（84日目）
⑥実治療	実治療開始-1日、1回目（0日目）、1日目、2回目（2日目）、3日目、3回目（4日目）、5日目	
⑦実治療：14日目、検査1（28日目）	14日目、検査1（28日目）	実治療1回目（0日目）～検査1（28日目）
⑧実治療：検査2（56日目）	検査2（56日目）	検査1（28日目）～検査2（56日目）
⑨実治療：70日目、検査3（84日目）	70日目、検査3（84日目）	検査2（56日目）～検査3（84日目）
⑩検査4（治験治療後1年目）	検査4（治験治療1年目）	検査4（治験治療1年目）の前28日以上
⑪検査5（実治療後1年目）	検査5（実治療1年目）	検査5（実治療1年目）の前28日以上
⑫中止時	中止時	前回Visitから中止時まで
予備	規定外にて観察・検査を実施した場合	なし

12.2 記入方法

・CRFの記載の記入及び訂正は治験責任医師又は治験分担医師が行う。治験責任医師又は治験分担医師は各被験者の各観察・検査が終了後、速やかにCRFを作成する。記入方法に関しては、「症例報告書の記載の手引き」に従う。

・治験協力者は、原資料が存在しその客観性が保証できる場合は、原資料からCRFに転記することが出来る。

・治験責任医師は、作成されたCRFについてその内容を点検し、確認した上で記名・押印又は署名を行う。

・治験責任医師は作成したCRFを定められた手順にて提出し、原本又は写しを保管する。

・治験責任医師は、CRFと原資料に矛盾がある場合には、その理由を説明する記録を作成の上定められた手順にて提出し、その写しを保管する。

・治験責任医師又は治験分担医師がCRFを訂正する場合、「症例報告書の記載の手引き」に従う。治験責任医師はCRFの変更又は修正の記録を定められた手順にて提出しその写しを保管する。
・CRFに記載されたデータのうち、下記の項目については治験責任医師又は治験分担医師がCRFへ直接記入する場合は、原資料の有無は不問とする。
1）有害事象の重篤性、治験機器との因果関係等のコメント
2）その他、CRF内のコメント
・治験ノートは、被験者が記載するものとし、治験責任医師又は治験分担医師が内容を確認後、写しをCRFと共に提出し、原本を保管する。

12.3 症例報告書の提出

治験責任医師は、CRFの原本及び治験ノートの写しを提出するとともに、CRF及び治験ノートの写しを東北大学臨床試験データセンターへモニターを介し提出する。提出先は下記とする。

CRFの送付先
東北大学病院　臨床試験データセンター
〒980-8574　宮城県仙台市青葉区星陵町1-1
TEL:022-717-7137　　　　FAX:022-717-7580
E-mail:dc033@crietodc.hosp.tohoku.ac.jp

12.4 原資料

原資料とは、治験の結果として症例報告書に記載されるデータのもとになる資料をいう。
(1) 被験者の同意及び情報提供に関する記録
(2) 診療録（検査伝票等を含む)、看護記録、被験者登録用紙など症例報告書作成の基となった記録
(3) 治験機器の使用に関する記録
(4) 治験ノート
(5) 各種画像検査データ

12.5 症例報告書中の記載内容を原資料とすべき項目の特定

症例報告書に記載されたデータのうち、以下に示す項目については、症例報告書の記載内容をもって原資料とする。
・患者背景における妊娠の有無
・合併症の有無
・併用薬の使用理由
・既往歴の有無
・治験機器の使用情報

・操作性評価、操作性に関するコメント
・有効性評価、有効性に関するコメント
・安全性に関するコメント
・併用薬・併用療法の有無
・中止・脱落の有無、理由、経過・転帰、コメント
・有害事象の有無、重篤性、処置の有無、転帰評価日、転帰、治験機器との因果関係の有無及びコメント

13. 治験の費用負担及び補償

13.1 利益相反について

当該治験は、2013 年 11 月～2016 年 3 月までは宮城県の補助金を、2016 年 4 月～2017 年 3 月までは国立研究開発法人日本医療研究開発機構委託研究開発費を、2017 年 4 月～7 月までは東北大学循環器内科関連経費を使用し、2017 年 8 月からは、国立研究開発法人日本研究開発機構(AMED)委託研究費の公的な資金を用いて、医師主導治験として実施する。

当該治験では、下川宏明ほかが株式会社日立製作所と共同研究を行い、開発した治験機器（超音波血管新生療法装置 T-75）を使用し、その有効性及び安全性を調べる。治験調整医師の下川宏明は、株式会社日立製作所等が寄付元である寄付講座の教員である。

当該治験は、東北大学の治験責任医師のグループにより公正に行う。当該治験における企業等との利害関係については、東北大学利益相反マネジメント委員会の審査と承認を得ている。また、当該治験における企業等との利害関係に追加・変更が生じた場合はその都度、東北大利益相反マネジメント委員会へ申告し審査を受けることにより、当該治験の企業等との利害関係についての公平性を保つ。

13.2 健康被害補償及び保険

当該治験の実施に起因して被験者に健康被害が発生した場合には、実施医療機関は治療その他必要な措置を講ずるものとし、実施医療機関及び被験者の故意もしくは重過失により生じた場合を除き、治験責任医師が適切な補償を行う。また、当該治験に起因する健康被害であって、賠償責任が生じた場合には、その原因を生じせしめた当事者（治験責任医師、実施医療機関など）の按分に沿って賠償金額を分担する。治験責任医師は、賠償責任及び補償責任の履行措置として、保険その他の必要な措置をとるものとする。

13.3 予定される治験費用及び被験者への支払い

治験期間中のすべての医療費は、被験者が負担する。治験責任医師は、当該治験に係る被験者の負担軽減費用について、実施医療機関と締結する契約書に基づき、定められた時期に実施医療機関に支払う。

14. 治験実施計画書の逸脱又は変更及び改訂

当該治験の実施に際しては、治験責任医師は、治験実施計画書案を実施医療機関の長を経由して治験審査委員会（IRB）に提出し、あらかじめ IRB の承認を経て実施医療機関の長の承認を得なければならない。

当該治験は治験責任医師と実施医療機関の長の合意のもとに当該治験実施計画書を遵守して実施する。

14.1 治験実施計画書の逸脱又は変更

(1) 治験責任医師又は治験分担医師は、治験実施医療機関の治験審査委員会（IRB）の事前の審査に基づく文書による承認を得ることなく、治験実施計画書からの逸脱又は変更を行わない。

(2) 治験責任医師又は治験分担医師は、被験者の緊急の危険を回避する等、医療上やむを得ない場合、IRB の事前の審査に基づく文書による承認を得ることなく、治験実施計画書からの逸脱又は変更を行うことができる。その際には、治験責任医師は、逸脱又は変更の内容及び理由を記載した文書を、直ちに治験実施医療機関の長に提出し、IRB の承認を得る。

14.2 治験実施計画書の改訂

(1) 治験責任医師は、治験の進行中に治験実施計画書に改訂の必要性が生じた場合は、その内容について、必要に応じて治験調整医師と協議する。

(2) 治験責任医師は、治験実施計画書、同意説明文書を改訂した場合は、治験実施計画書改訂版、同意説明文書改訂版を速やかに治験実施医療機関の長に提出する。

(3) 治験責任医師は、IRB の承認を経て実施医療機関の長の承認を得た後に、治験実施計画書改訂版の内容ならびに当該同意説明文書改訂版を遵守して治験を行わなければならない。

15. 治験の中止・中断・終了

治験の一部及び全体の中止・中断規定は以下のとおりとし、いずれの場合も中止時点の成績の収集及び解析を行う。なお、個々の被験者の中止基準については、「4.3 中止基準」に記載した。

15.1 治験の中止・中断

当該治験機器の開発の妥当性が否定された場合及び予期せぬ重篤な有害事象が認められ治験の実施が医学的・倫理的に実施不可能と判断された場合等、当該治験の進行中に治験全体を中止又は中断せざるを得ない理由が生じた場合、治験責任医師は治験調整医師と協議し、治験の中止・中断及びその理由の詳細を実施医療機関の長及び規制当局、臨床試験データセンター、臨床研究推進センターに速やかに通知する。実施医療機関の長は治験審査委員会に

その旨を通知し、文書で詳細を説明する。治験責任医師又は治験分担医師は、被験者にその旨を通知し、適切な治療及び事後処理を保証する。

15.2 治験の終了

治験が終了した場合には、治験責任医師は実施医療機関の長にその旨を文書で通知し、治験結果の概要を文書(治験終了報告書)で報告する。実施医療機関の長は、治験審査委員会に対し、速やかに終了の旨を文書で通知するとともに、治験終了報告書に基づき、治験結果の概要を報告する。

16. 効果安全性評価委員会について

効果安全性評価委員会は、治験の継続の適否又は治験実施計画書の変更について審議することを目的として、自ら治験を実施する者によって設置され、別途定められた手順に従い、治験の進行、安全性データ及び重要な有効性エンドポイントを評価するものである。

17. 中央判定委員会について

中央判定員会は、有効性評価の副次的評価項目に関する評価を行うことを目的として設置され、別途定められた手順書に従い、治験責任医師及び治験分担医師以外の者が治験実施後に得られた各種データを客観的に評価・解析するものである。

18. 記録等の保存

(1) 実施医療機関の長は、実施医療機関において保存すべき治験に係る文書又は原資料を、次の①又は②のうちいずれか遅い日まで保存する。ただし、治験責任医師又は実施医療機関の長がこれよりも長期間の保存を必要とする場合には、保存期間及び保存方法について治験責任医師又は実施医療機関の長と協議する。

① 当該治験機器に係る製造販売承認日（開発が中止された場合には開発中止が決定された日から 3 年が経過した日）

② 治験の中止又は終了後 3 年が経過した日

(2) 治験審査委員会の設置者は、業務手順書、委員名簿（各委員の資格を含む)、委員の職業及び所属のリスト、提出された文書、会議の記録、その概要及び書簡等の記録を次の①又は②のうちいずれか遅い日まで保存する。ただし、治験責任医師がこれよりも長期間の保存を必要とする場合には、保存期間及び保存方法について治験責任医師と協議する。

①当該治験機器に係る製造販売承認日（開発が中止された場合には開発中止の連絡を受けた日から 3 年が経過した日）

② 治験の中止又は終了後 3 年が経過した日

(3) 治験責任医師は、保存すべき治験に係る文書又は記録を、次の①又は②のうちいずれか遅い日までの間保存する。

① 当該治験機器に係る製造販売承認日から 5 年が経過した日（開発が中止された場合には開発中止が決定された日から 3 年が経過した日）又は再審査が終了する日

② 治験の中止又は終了後 3 年が経過した日

19. 公表に関する取決め

治験責任医師又は治験分担医師は、当該治験により得られた情報を学会等外部に公表しようとする場合には、あらかじめ文書により治験調整医師の承諾を得るものとする。

20. 治験実施体制

当該治験の実施体制については、別紙 1 に示した。

21. 参考文献

1. Nishida T, Shimokawa H, Oi K, Tatewaki H, Uwatoku T, Abe K, Matsumoto Y, Kajihara N, Eto M, Matsuda T, Yasui H, Takeshita A, Sunagawa K. Extracorporeal cardiac shock wave therapy markedly ameliorates ischemia-induced myocardial dysfunction in pigs in vivo. Circulation. 110:3055-61, 2004.
2. Fukumoto Y, Ito A, Uwatoku T, Matoba T, Kishi T, Tanaka H, Takeshita A, Sunagawa K, Shimokawa H. Extracorporeal cardiac shock wave therapy ameliorates myocardial ischemia in patients with severe coronary artery disease. Coron Artery Dis. 17:63-70, 2006.
3. Kikuchi Y, Ito K, Ito Y, Shiroto T, Tsuburaya R, Aizawa K, Hao K, Fukumoto Y, Takahashi J, Takeda M, Nakayama M, Yasuda S, Kuriyama S, Tsuji I, Shimokawa H. Double-blind and placebo-controlled study of the effectiveness and safety of extracorporeal cardiac shock wave therapy for severe angina pectoris. Circ J. 74:589-591, 2010.
4. Ito K, Fukumoto Y, Shimokawa H. Extracorporeal shock wave therapy for ischemic cardiovascular disorders. Am J Cardiovasc Drugs. 11:295-302, 2011.
5. Barzelai S, Sharabani-Yosef O, Holbova R, Castel D, Walden R, Engelberg S, Scheinowitz M. Low-intensity ultrasound induces angiogenesis in rat hind-limb ischemia. Ultrasound Med Biol. 32:139-5, 2006.
6. Mizrahi N, Seliktar D, Kimmel E. Ultrasound-induced angiogenic response in endothelial cells. Ultrasound Med Biol. 33:1818-29, 2007.
7. Ramli R, Reher P, Harris M, Meghji S. The effect of ultrasound on angiogenesis: an in vivo study using the chick chorioallantoic membrane. Int J Oral Maxillofac Implants. 24:591-6, 2009.
8. Hanawa K, Ito K, Aizawa K, Shindo T, Nishimiya K, Hasebe Y, Gao JY, Hasegawa H, Yasuda S, Kanai H, Shimokawa H. Low-intensity pulsed ultrasound induces angiogenesis and ameliorates left ventricular dysfunction in a porcine model of chronic myocardial ischemia. 2012 年米国心臓協会年次集会で発表（2012 年 11 月 ;ロサンゼルス）。
9. Burkhoff D, Schmidt S, Schulman SP, Myers J, Resar J, Becker LC, Weiss J, Jones JW, the ATLANTIC Investigators. Transmyocardial laser revascularisation compared with continued

medical therapy for treatment of refractory angina pectoris: a prospective randomised trial Lancet. 354:885-90, 1999.

10. Losordo DW, Henry TD, Davidson C, Lee JS, Costa MA, Bass T, Mendelsohn F, Fortuin FD, Pepine CJ, Traverse JH, Amrani D, Ewenstein BM, Riedel N, Story K, Barker K, Povsic TJ, Harrington RA, Schatz RA, the ACT34-CMI Investigators. Intramyocardial, autologous CD34+ cell therapy for refractory angina. Circ Res. 109:428-436, 2011.
11. Hare JM, Fishman JE, Gerstenblith G, Difede Velazquez DL, Zambrano JP, Suncion VY, Tracy M, Ghersin E, Johnston PV, Brinker JA, Breton E, Davis-Sproul J, Schulman IH, Byrnes J, Mendizabal AM, Lowery MH, Rouy D, Altman P, Wong Po Foo C, Ruiz P, Amador A, Da Silva J, McNiece IK, Heldman AW. Comparison of allogeneic vs autologous bone marrow–derived mesenchymal stem cells delivered by transendocardial injection in patients with ischemic cardiomyopathy: The POSEIDON randomized trial. JAMA, 2012. Published online November 6, 2012.
