# Supplementary material for: A randomized, double-blind, placebo-controlled pilot trial of low-intensity pulsed ultrasound therapy for refractory angina pectoris
Source: PLoS One. 2023 Jun 23;18(6):e0287714. doi: 10.1371/journal.pone.0287714 (PMC10289346; doi:10.1371/journal.pone.0287714)
Supplement: S4 File — (DOCX) [file pone.0287714.s008.docx]

1-1 Seiryo-machi

Aoba-ku, Sendai

980-8574 JAPAN

TEL: +81-22-717-7153

FAX: +81-22-717-7156

A Clinical Trial to Evaluate Effectiveness and Safety

of an Ultrasound Apparatus T-75

for the Treatment of Patients with Refractory Angina Pectoris

Principal investigator:

Hiroaki Shimokawa

Professor and Chairman

Department of Cardiovascular Medicine

Tohoku University Graduate School of Medicine

1. **Introduction**

Ischemic heart disease (IHD) is one of the major causes of death in developed countries, and its morbidity is also increasing in developing countries. Although recent advances in therapeutic strategies have reduced the mortality of patients with IHD, the number of severe IHD patients is increasing as the population is rapidly aging. Thus, non-invasive therapeutic strategies for severe IHD remain to be developed. We have previously demonstrated that low-energy extracorporeal cardiac shock wave (SW) therapy improves myocardial ischemia in a porcine model of chronic myocardial ischemia and patients with severe angina pectoris.

Ultrasound is a form of sound whose frequency is higher than the natural audible range for humans (>20 kHz) and ultrasonography has been widely used as diagnostic devices for several decades. In addition to diagnostic purposes, ultrasound is clinically used for therapeutic applications, including tumor ablation, thrombolysis, bone regeneration, and facilitated drug delivery. We have recently reported that the low-intensity pulsed ultrasound (LIPUS) therapy induces angiogenesis in the ischemic myocardium and normalizes myocardial function in a porcine model of chronic myocardial ischemia in vivo. Here, we aimed to evaluate the effectiveness and safety in patients with refractory angina pectoris.

Ref.) Hanawa K, Ito K, Aizawa K, Shindo T, Nishimiya K, Hasebe Y, Tuburaya R,

Hasegawa H, Yasuda S, Kanai H, Shimokawa H.

Low-intensity pulsed ultrasound induces angiogenesis and ameliorates left

ventricular dysfunction in a porcine model of chronic myocardial ischemia.

PLoS One. 2014;9:e104863.

**2. Study Objectives**

The objective of this study is to evaluate the effectiveness and safety of the ultrasound therapeutic device and to improve quality of life (QOL) in patients with refractory angina pectoris

**3. Study Design**

**3.1 Study design**

A prospective multicenter, randomized, double-blind, placebo-controlled study.

The goal of this study is to improve the quality of life (QOL) of patients by developing new treatments for ischemic heart disease. Specifically, we applied ultrasound therapy for patients with ischemic heart disease, and the average weekly frequency of use of nitrates, subjective symptoms, exercise tolerance, improvement of myocardial blood flow, and cardiac function, and major cardiovascular events (Major) Adverse Cardiac Events; MACE) (cardiovascular death, non-fatal myocardial infarction, unstable angina / heart failure requiring hospitalization) is evaluated.

**3.2 Study population**

All subjects with ischemic heart disease who meet inclusion criteria and do not meet exclusion criteria will be randomly assigned to either treatment group or placebo group.

Inclusion criteria

1) Informed consent in writing.

2) Men and women 20 years of age or older.

3) Patients who are able to be admitted to a hospital for at least 6 days.

4) Patients with refractory angina pectoris who have no indication of revascularization

(PCI or CABG) or in whom the risk of revascularization therapy does not meet the

expected improvement.

5) Patients who have chest pain even under standard medical therapy and use at least

one use of nitrates per week.

6) Evidence of myocardial ischemia with myocardial stress-scintigraphy.

7) Able to fill out a trial notebook for recording the frequency of nitrate use and chest

pain.

Exclusion criteria

1) Patients whose target area cannot be observed with echocardiography.

2) Intracardiac thrombus.

3) Less than 28 days after the last revascularization (PCI or CABG).

4) Q-wave myocardial infarction within 84 days.

5) Non-Q-wave myocardial infarction within 42 days.

6) Cardiogenic shock or worsening heart failure (to require continuous infusion of

cardiovascular agents such as inotropic agents or vasodilator ).

7) Patients whose clinical condition changed after the last coronary angiography.

8) Uncontrolled diabetic retinopathy.

9) Patients who have malignant tumor or who have undergone surgery because of

malignancy within the past five years.


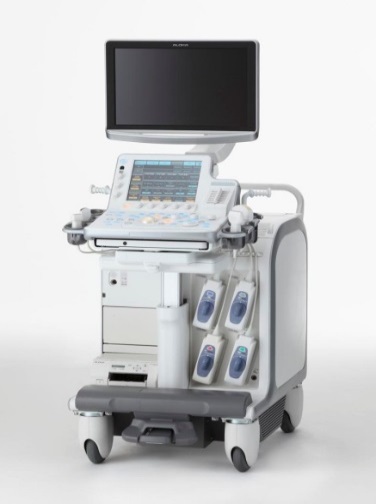
**3.3 A therapeutic device**

For the LIPUS treatment, we use an ultrasound device (T-75; HITACHI Aloka Medical, Ltd., Mitaka, Japan).

**3.4 Therapeutic conditions**

・Irradiation with pulse wave in B mode

・Intensity : 0.25 W/cm^2^

・Frequency : 1.875 MHz

・Number of cycles : 32

(To see Fig.1 of Hanawa K, et al. PLoS One. 2014)

・Ultrasonic irradiation to the heart for 20 minutes at 3 different short-axis levels


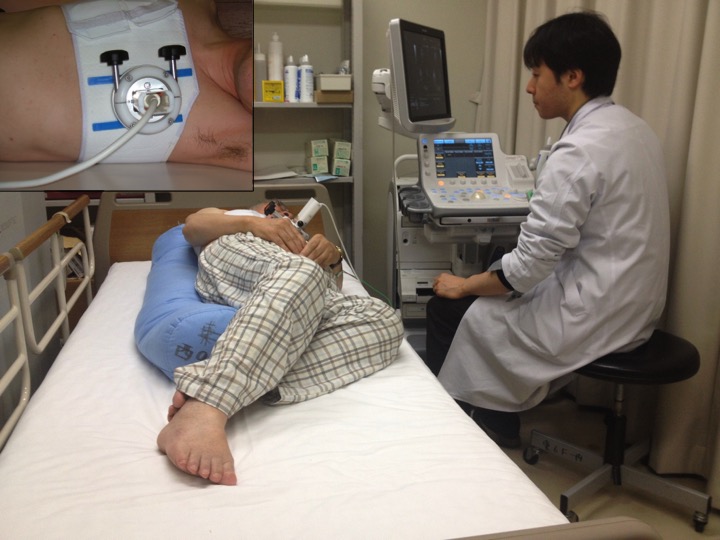
with a 5-minute interval between each irradiation

Treatment procedure

After registration, the subjects and evaluators were blinded and treated with this study device. It was decided that examinations and observations for primary endpoints would be conducted 84 days after the initial clinical trial procedure, and then actual treatment would be performed within 28 days. In both the clinical trial procedure and the actual treatment, ultrasonic irradiation was performed three times a day as one set, and a total of three sets were performed every other day. The placebo-treated group did not receive ultrasound during the trial treatment.

- Treatment method

1) Connect the probe to the main body of the ultrasonic diagnostic equipment.

2) Point the monitor toward the operator with the monitor handle so that it cannot be seen by the subject.

3) Operate the power switch on the operation panel to turn on the power. Log in and enter the password to make it operable.

4) From the ID input screen, use the keyboard to enter the registration number in the Patient ID field and the operator's name in the Sonographer field.

5) Wear a fixed belt on the subject's chest so that the probe does not move during treatment and the irradiation site does not shift. The fixation belt is adjusted so that the range of motion of the probe can be observed in the entire left ventricle. The fixed belt is selected from S, M, L, and LL sizes according to the subject's physique.

6) An acoustic medium is applied to the test site of the subject or the contact surface of the probe, and the positioning probe is attached to the fixing belt with the positioning probe attached to the fixture, and an image of the affected area to be treated is displayed.

**3.5 Endpoints**

(1) Efficacy

1) Primary endpoint

・Nitroglycerin use per week (sublingual tablets, sprays)

2) Secondary endpoints

・Symptom (CCS class score, frequency of angina episodes per week)

・Exercise tolerance test (treadmill exercise ECG, six-minute walk test)

・Myocardial perfusion (adenosine stress myocardial perfusion scintigraphy)

・Left ventricular ejection fraction (LVEF) in patients with baseline LVEF < 40%

(UCG, cardiac MRI)

(2) Safety

1) Clinical laboratory examination

2) Chest X-ray

3) Frequency of major cardiovascular events one year after first treatment (cardiovascular death, nonfatal myocardial infarction, hospitalization due to unstable angina or heart failure)

4) Adverse events

5) Malfunction of the ultrasound device

**
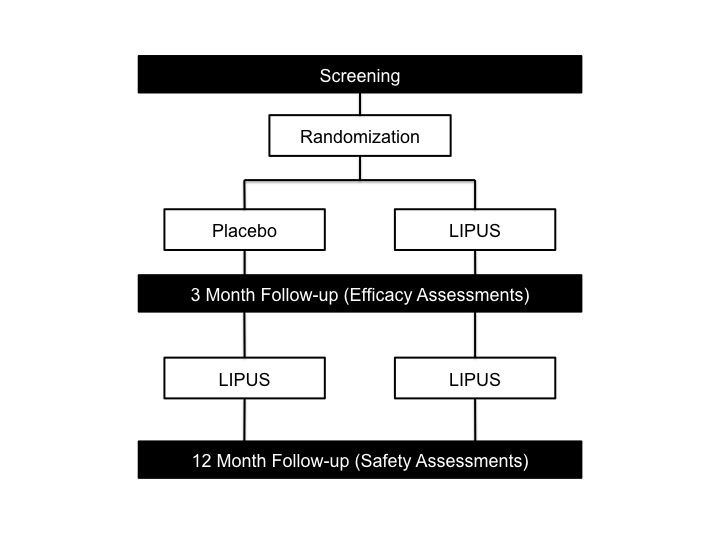
**

**4. Statistical analysis**

**4.1 Effectiveness analysis**

Primary endpoints

(1) Endpoint

Changes in nitrate usage frequency before treatment (28 days immediately before registration) and after treatment (28 days 57 to 84 days after the first clinical trial treatment)

(2) Method

The average weekly frequency of use of nitrates before and after treatment (average frequency of use per week calculated from the frequency of use for 28 days) was evaluated.

Regarding the weekly mean frequency of use, the amount of change from before treatment was calculated, the mean value of each group, the difference between groups of mean values, and the 95% confidence interval were estimated, and the comparison between groups was performed using the t-test. The significance level was 5% on both sides. In addition, the median weekly average frequency of use and the number and proportion of subjects whose average weekly frequency of use improved after treatment compared to before treatment were also tabulated for each group.

Secondary endpoints

In all cases, the significance level was set to 5% on both sides.

1. CCS class score

(1) Endpoints

Changes in CCS class score before treatment (between consent and registration) and after treatment (56 to 84 days after initial clinical trial treatment and actual treatment)

(2) Method

The mean and 95% confidence intervals for each group were calculated for the pre- and post-treatment values ​​of the CCS class score. In addition, the mean value of each group, the difference between the mean values, and the 95% confidence interval were estimated for the amount of change from before treatment, and the t-test was used to make a comparison between the groups. In addition, the number and proportion of subjects whose CCS class score improved by 1 point or more after treatment compared to before treatment were also tabulated for each group and the difference in proportion was tested.

2. Average weekly frequency of chest pain and average weekly use of nitrates

(1) Endpoints

Weekly average frequency of chest pain before treatment (28 days immediately before registration), after treatment (28 days 57 to 84 days after the first clinical trial treatment and actual treatment), and before treatment of average weekly frequency of nitrate use (registration) Changes between (28 days immediately before) and after treatment (28 days from 57 days to 84 days after the first actual treatment)

(2) Method

For the weekly average frequency of chest pain before and after treatment (average frequency per week calculated from the frequency of occurrence for 28 days), the mean value for each group and the 95% confidence interval were calculated. In addition, the mean value of each group, the difference between the mean values, and the 95% confidence interval were estimated for the amount of change from before treatment, and the t-test was used to make a comparison between the groups. In addition, the number and proportion of subjects whose median and weekly average frequency of occurrence improved after treatment compared to before treatment were calculated. For the number and proportion of subjects who improved, the difference in proportion was tested.

In addition, a comparison of the average weekly frequency of nitrate use before and after treatment during the actual treatment period was also analyzed by the same method.

3. Treadmill exercise electrocardiogram

(1) Endpoints

Exercise endurance: Treadmill exercise ECG exercise load, exercise load per unit time, and total load time before treatment (from consent acquisition to immediately before the first clinical trial treatment) and after treatment (first clinical trial treatment and actual treatment) 84 days later)

(2) Method

The mean values and 95% confidence intervals for each group were calculated for the exercise load, exercise load per unit time, and total load time before and after treatment on the treadmill exercise electrocardiogram. In addition, the mean value of each group, the difference between the mean values, and the 95% confidence interval were estimated for the amount of change from before treatment, and the t-test was used to compare between the groups.

4. 6-minute walking test

(1) Endpoints

Changes in walking distance of the 6-minute walking test before treatment (from obtaining consent to immediately before the first implementation of clinical trial treatment) and after treatment (84 days after the initial implementation of clinical trial treatment and actual treatment)

(2) Method

The mean and 95% confidence intervals for each group were calculated for the pre- and post-treatment values of the 6-minute walk. Regarding the amount of change from before treatment, the mean value of each group, the difference between the mean values, and the 95% confidence interval were estimated, and the t-test was used to compare between the groups.

5. Stress myocardial scintigraphy

(1) Endpoints

Changes in Summed difference Score (SDS) and ischemic dose in stress myocardial scintigraphy using adenosine before treatment (from consent acquisition to screening) and after treatment (examination 84 days after clinical trial treatment and initial treatment)

(2) Method

The mean values and 95% confidence intervals for each group were calculated for the pre- and post-treatment values of SDS and ischemia in stress myocardial scintigraphy using adenosine. In addition, the mean value of each group, the difference between the mean values, and the 95% confidence interval were estimated for the amount of change from before treatment, and the t-test was used to compare between the groups.

6. Echocardiography

(1) Endpoints

Cardiac function in patients with low cardiac function with a left ventricular ejection fraction of 40% or less: Changes in the following items of echocardiography before and after treatment (at the time of screening) and after treatment (84 days after the initial treatment and actual treatment)

・ Presence or absence of changes in the ischemic region and changes in heart movement (abnormal wall motion)

・ Left room ejection fraction

(2) Method

The mean value and 95% confidence interval for each group were calculated for the pre- and post-treatment values of the left ventricular ejection fraction of echocardiography. In addition, the mean value of each group, the difference between the mean values, and the 95% confidence interval were estimated for the amount of change from before treatment, and the t-test was used to compare between the groups. Regarding the appearance of changes in the ischemic region and changes in heart movement, changes from before treatment, after treatment, and before treatment were frequently tabulated.

7. Cardiac MRI

(1) Endpoints

Cardiac function in patients with hypocardiac function with a left ventricular ejection fraction of 40% or less: Changes before cardiac MRI treatment (from consent acquisition to immediately before the first clinical trial treatment) and after treatment (84 days after the first clinical trial treatment and actual treatment)

・ Left room ejection fraction

・ Left ventricle stroke volume

(2) Method

The mean values and 95% confidence intervals for each group were calculated for the left ventricular ejection fraction and the left ventricular stroke volume before and after treatment for cardiac MRI. In addition, regarding the amount of change from before treatment, the mean value of each group, the difference between the mean values, and the 95% confidence interval were estimated and compared between the groups using the t-test.

8. Analysis of safety endpoints

(1) Adverse events

1) Endpoints

・ Status of adverse events

・ Status of occurrence of adverse events by severity

・ Withdrawal rate due to adverse events

2) Method

All adverse events that occurred in the subjects, operators, etc. from the start of treatment to the end of the clinical trial were included in the total. NCI CTCAE v4.0 was used to evaluate adverse events.

-For adverse events, the number of occurrences, the number of cases of occurrence, and the incidence rate were calculated and summarized for each group.

・ Regarding adverse events, the number of occurrences was calculated by severity and period (clinical trial treatment period / actual treatment period) and summarized for each group.

・ With the discontinuation due to an adverse event as an event, the withdrawal rate curve in each group was estimated by the Kaplan-Meier method and compared between the groups by the logrank test. Point estimates and 95% confidence intervals for adverse event withdrawal rates were calculated for each group one year after the initial treatment.

(2) Malfunction

1) Endpoints

Occurrence of malfunction

2) Method

All defects that occurred in the clinical trial equipment during the clinical trial period were counted, and the number of occurrences was calculated for each time of occurrence (at the time of clinical trial, others, etc.). A clinical trial treatment period analysis was performed on the defects that occurred during irradiation of the subjects during the clinical trial treatment period. The period was up to the clinical trial treatment observation period examination 3 (84 days after the first clinical trial treatment), and the later date of the ultrasonic examination and the 6-minute walking test was used.

(3) MACE

1) Endpoints

Expression of MACE up to 1 year after the first actual treatment

2) Methods

The number, number, and proportion of cases of MACE up to 1 year after the first actual treatment were calculated. It was also confirmed that the frequency did not exceed 40%. Furthermore, an indirect comparison was made with past paper reports targeting heart disease cases similar to the study. In addition, the background information of past paper reports was confirmed and compared and considered with the population of this study.

**4.2 Definition of adverse events**

Adverse events (including MACE) are all unfavorable or occurring in subjects and operators who have undergone treatment with the study device (including placebo treatment), regardless of whether or not there is a causal relationship with the study device. It was defined as an unintended disease or disorder and its signs (including abnormal laboratory test values).

Adverse events were collected during the period from the treatment with the study device to the end or discontinuation of the observation period of each subject (excluding the follow-up period).

- Description of adverse events

As a general rule, the name of the adverse event is described in the case report form (CRF) with the name of the diagnosis and the name of the disease (disease name). If the diagnosis / disease name cannot be specified, or if the investigator or investigator determines that it is appropriate not to use the diagnosis / disease name, the clinical symptom or sign (including abnormal laboratory test values) is used as the adverse event name.

The investigator or investigator may consider the name of the adverse event, its degree (serious, non-serious), the reason for determining it to be serious, the date of occurrence, the date of outcome, the treatment, and the outcome (recovery,) for all adverse events that occurred. The symptoms improved, recovered but had aftereffects, not recovered, died), the causal relationship with the study device, and comments (reasons for determining the causal relationship with the study device, etc.) were described in detail in the adverse event column of the CRF.

**4.3 Randomisation method**

CCS class score ("II", "III or IV"), frequency of nitro use per week ("4 times / week or less", "5 times / week or more"), left ventricular ejection fraction ("40% or more") , "Less than 40%"), and the implementing medical institution was used as an allocation factor, and the patients were randomly stratified into two groups, the actual treatment group and the placebo treatment group.

The allocation table was created by the person in charge of statistical analysis using the statistical analysis software SAS Proc Plan. A random number seed of 4328 was used. For the combination of allocation factors other than facilities, we created allocation tables for 40 people and 10 facilities, respectively. A and P in the allocation table represent the actual treatment group and the placebo treatment group, respectively, and the registration number is added.

**5 Ethics**

**5.1 Matters concerning obtaining consent of subjects**

We decided to obtain consent by following the procedure below.

(1) The investigator should fully explain to the subject using an explanatory document approved by the IRB prior to participating in the clinical trial, and then voluntarily consent to participate in the clinical trial in writing.

(2) In the explanation, the investigator gives the subject an opportunity to ask questions and the time required to decide whether or not to participate in the clinical trial before obtaining consent from the subject. In addition, the investigator or collaborator as a supplementary explainer will answer all questions to the satisfaction of the subject.

(3) The consent document shall be signed, stamped or signed by the investigator who gave the explanation, and the subject, and each person shall enter the date. If the clinical trial collaborator gives a supplementary explanation, the clinical trial collaborator will also sign, seal, or sign and enter the date.

(4) The investigator gives a copy of the consent document with the name, seal or signature and date, and other explanatory documents to the subject before the subject participates in the clinical trial.

(5) The original consent document shall be retained at the implementing medical institution.

**5.2 Revision of explanatory document / consent document**

(1) When new important information that may be related to the consent of the subject (usually information that requires revision of the explanatory document/consent document) is obtained, the investigator will promptly explain based on the information. It was decided to revise the document / consent document and obtain IRB approval.

(2) The investigator will explain again using the revised explanatory document/consent document, and will obtain written consent from the subjects regarding the continuation of participation in the clinical trial. The investigator decided to give the subject a copy of the consent document with a new name, seal or signature and date, and an explanatory document. The original consent document will be retained at the implementing medical institution.

**5.3 Matters concerning the protection of human rights of subjects**

In selecting a subject, the investigator shall determine the subject's health condition, symptoms, age, consent ability, and dependency with the investigator based on the selection criteria and exclusion criteria from the viewpoint of human rights protection. We carefully examined the suitability of requesting participation in clinical trials, taking into consideration the presence or absence of participation in other clinical trials.

Subject registration and identification of the subject in the CRF are performed by the subject identification number, and the subject's name are used for direct viewing of the source materials related to the clinical trial and the subject's explanatory documents and consent documents, and for the publication of the clinical trial results. Sufficient consideration was given to protecting the privacy of diseases.

**6. Clinical trial quality control and quality assurance**

**6.1 Monitoring**

The monitoring staff appointed by the clinical trial coordinator confirms that the clinical trial is being conducted in accordance with all the contents described in the clinical trial protocol and the medical device GCP according to the “monitoring procedure manual” prepared by the clinical trial coordinating doctor. Therefore, monitoring with the implementing medical institution was carried out regularly.

**6.2 Audit**

The person in charge of auditing appointed by the clinical trial coordinating doctor conducted the audit at the conducting medical institution and the outsourced development business in accordance with the “Audit Procedure Manual” prepared by the clinical trial coordinating doctor.

**6.3 Direct viewing of original materials.**

The director of the conducting medical institution and the investigator will accept monitoring, auditing, and investigations by IRBs and domestic and overseas regulatory agencies, and will make all clinical trial-related records, including sources, available for direct viewing (including copying). For details, follow the standard business procedure manual. The subject was allowed to browse directly by signing, stamping, or signing the consent document, which was explained using the explanatory document.

**7 Clinical trial procedure**

**7.1 Obtaining consent**

(1) When selecting a subject, the investigator considers the subject's health condition, symptoms, age, gender, consent ability, participation in other clinical trials, etc., and targets the subject in the clinical trial. Carefully consider the suitability of this.

(2) The investigator shall fully explain the clinical trial to the subjects who are judged to be appropriate for the clinical trial, and obtain written consent.

**7.2 Screening**

It was decided to carry out the necessary inspections and surveys from the acquisition of consent to the time of registration. In addition, clinical trial notes will be distributed to the subjects at the time of consent acquisition, and the number of times the subject will use nitrates/day and the number of chest pains/day will be 28 days or more (from the day after consent acquisition to the day before registration) from the consent acquisition date to the scheduled registration date.

**7.3 Judgment of suitability of selection criteria by the Adaptation Evaluation Committee**

The study coordinating physician has set up an indication evaluation committee for the purpose of determining suitability for selection criteria.

The Adaptation Evaluation Committee is independent of those who conduct clinical trials, clinical trial coordinating doctors, clinical trial coordinating secretariats, directors of clinical trial institutions, and persons involved in conducting clinical trials (including support staff) in Japan. It consisted of a total of two members, one specialist from the Cardiovascular Surgery Society and one specialist from the Japan Cardiovascular Intervention Therapy Society.

The Adaptation Evaluation Committee evaluated the adaptation of the subject data sent by the investigator to the selection criterion based on the following evaluation criteria. If the evaluation results of two members are both qualified, it is judged to be "qualified", and if the evaluation results of either one or both members are not qualified, it is judged to be "not qualified". The evaluation results will be reported to the investigator by fax.

<Evaluation criteria>

・ Specialist of the Japanese Society of Cardiovascular Surgery: Diagnosis of ischemic heart disease that is resistant to drug therapy for ischemic heart disease at the time of registration and is difficult to treat by coronary artery bypass surgery or has a high risk compared to its benefits. Patient.

・ Specialist of the Japanese Society of Cardiovascular Intervention Therapy: Resistance to drug therapy for ischemic heart disease at the time of registration, difficult to treat by percutaneous coronary intervention, or high risk compared to benefits Patients diagnosed with bloody heart disease.

**7.4 Subject registration and allocation of clinical trial treatment groups**

(1) The investigator should refer to the suitability of the selection criteria by the Adaptation Evaluation Committee, the clinical trial notes (statement rate of 70% or more) described by the subject for 28 days or more, and the selection criteria and exclusion criteria. Based on this, the eligibility of the subject was determined and a subject identification number was assigned.

(2) The investigator fills out the required items on the subject registration form and faxes it to the registration center together with the eligibility judgment results of the Adaptation Evaluation Committee from 14 days to 2 days before the first clinical trial treatment. It is stipulated that the sending may be done by the investigator under the guidance.

(3) The registration center confirmed the eligibility and allocation factor from the subject registration form. If eligible, the registration number next to the assigned treatment group was entered on the eligibility confirmation contact form from the allocation table and faxed to the investigator or investigator. In addition, a copy of the eligibility confirmation contact form and the subject registration form was sent to the clinical trial coordinating physician.

(4) The investigator who has been appointed as the investigator in advance confirms the registration number from the investigator who received the eligibility confirmation contact form, and registers it from the pre-distributed allocation table. The allocation group assigned to the number (actual treatment group, placebo treatment group) was confirmed.

(5) If the selection criteria are not met or the exclusion criteria are violated and the registration is not achieved, the clinical trial sharing form will be sent by the investigator stating that the patient is ineligible and the reason for the ineligibility. Cases that became ineligible were disqualified and the study was terminated.

(6) The investigator must not start the treatment prescribed in this study until the registration of subjects is completed. Post-registration and double registration are not allowed. If post-registration or double registration is found, the investigator will promptly contact the registration center.

**7.5 Blinding procedure**

(1) Nomination of a doctor in charge of clinical trial treatment at the implementing medical institution

At the conducting medical institution, the investigator appoints the investigator in charge of clinical trial treatment. The investigators in charge of clinical trial treatment were selected from the investigators. Only the investigator in charge of clinical trial treatment can know the allocation group (actual treatment group, placebo treatment group) of the subjects in the medical institution.

In addition, the doctor in charge of clinical trial treatment will only carry out clinical trial treatment, and will not be able to perform tasks that affect clinical trial results other than clinical trial treatment (examination of subjects, description of CRF, judgment of test results, etc.). In addition, the doctor in charge of clinical trial treatment nominated a person who confirms that the clinical trial treatment is being performed properly from among the medical staff as much as possible. The nominated health care workers will be able to know the allocation group.

(2) Storage of allocation table

The allocation table is a procedure owned by the registration center and the investigator. It is possible to view only the investigator and the medical staff appointed by the investigator at the conducting medical institution, and cannot disclose it to others (the investigator, the subject, other medical staff, etc.). In addition, the allocation table was kept in a storage that can be unlocked only by the investigator in charge of clinical trial treatment until the permission of the investigator.

(3) Clinical trial treatment

All clinical trial treatments performed at the conducting medical institution were carried out by the investigator. Except in an emergency, the investigator, and other medical personnel are not allowed to be present during the clinical trial treatment. The medical staff appointed by the investigative treatment doctor will be able to attend.

(4) Key open

In principle, the key opening of the allocation group (actual treatment group, placebo treatment group) of each subject was decided to be performed according to the instruction of the study coordinating doctor after the clinical trial was completed and the case data of all the conducting medical institutions were fixed.

Even during the clinical trial period, if key opening is required such as in an emergency, it will be possible to know the allocation group (actual treatment group, placebo treatment group) of each subject according to the instruction of the investigator. In that case, the investigator decided to disclose only the assigned group of the applicable subjects. In the event of an emergency key opening, the investigator will report the situation as much as possible in advance and explain the reason for the key opening.
